# Stem cells ameliorate neurotrauma-induced visual disturbances and retinal degeneration via broad normalization of β-catenin-related signaling

**DOI:** 10.7150/ijms.123975

**Published:** 2026-01-23

**Authors:** Shu-Chun Kuo, Chung-Hsin Tseng, Suan Hwang, Chia-Yi Lee, Ting-Feng Wu, Pi-Yu Chao, Anthony Lu, Ching-Ping Chang, Chung-Ching Chio

**Affiliations:** 1 Department of Ophthalmology, Chi Mei Medical Center, Tainan, Taiwan.; 2 Department of Optometry, Chung Hwa University of Medical Technology, Tainan, Taiwan.; 3 Department of Medical Research, Chi Mei Medical Center, Tainan 710, Taiwan.; 4 Department of Biotechnology and Food Technology, Southern Taiwan University of Science and Technology, Tainan 710, Taiwan.; 5 Department of Medical Research, Chi Mei Medical Center, Tainan 710, Taiwan.; 6 Division of Neurosurgery, Department of Surgery, Chi Mei Medical Center, Tainan 710, Taiwan.

**Keywords:** traumatic brain injury, retinal degeneration, mesenchymal stem cell, β-catenin

## Abstract

**Background:**

Traumatic brain injury (TBI) is associated with visual dysfunction and retinal degeneration, but the underlying mechanisms and therapeutic options remain limited. Mesenchymal stem cells (MSCs) have shown neuroprotective effects in various central nervous system injuries, including optic neuropathies.

**Objective:**

To investigate the protective effects and mechanisms of MSC therapy in TBI-induced retinal degeneration using *in vivo* and *in vitro* models.

**Methods:**

Repeated mild TBI was induced in adult male Wistar rats by lateral fluid percussion. On day 3 post-injury, rats received intravenous MSCs (4 × 10⁶ cells/ml/kg) or saline. Visual and neurological functions were assessed using the visual cliff test and modified neurological severity score (mNSS). Thirty-five days after TBI, retinal tissues were collected for histological and immunofluorescence analysis. *In vitro*, R28 retinal precursor cells underwent stretch injury (SI) and were then cocultured with MSCs. Cell viability, apoptosis, mitochondrial membrane potential, reactive oxygen species (ROS), and β-catenin signaling were evaluated.

**Results:**

TBI caused visual deficits, brain injury, retinal ganglion cell loss, thinning of the ganglion cell complex, increased neuronal apoptosis, and decreased β-catenin-positive neurons. *In vitro*, SI led to decreased cell viability, increased apoptosis and autophagy, mitochondrial dysfunction, ROS overproduction, and reduced β-catenin expression. MSC treatment ameliorated both *in vivo* and *in vitro* injuries, restoring visual function, preserving retinal structure, and normalizing β-catenin-related pathways.

**Conclusion:**

MSC therapy mitigates TBI-induced visual dysfunction and retinal degeneration, in association with β-catenin-related neuroprotective signaling, and may represent a promising strategy for treating TBI-related visual and retinal injury.

## Introduction

Repeated traumatic brain injury (TBI) causes neuroinflammation, glial cell activation, and progressive cognitive impairment 1, 2. In addition to these neurological consequences, TBI can induce structural damage in brain regions involved in visual processing, leading to visual disturbances and dysfunction 3-7. The spectrum of post-TBI visual impairments includes photophobia, double vision, and vision loss, which substantially impact quality of life 7. Experimental studies have demonstrated that mice with multiple TBIs exhibit impaired performance in the visible platform water maze and make increased errors in the visual cliff test 8. Furthermore, histological analyses of the optic tract in TBI models have revealed glial cell activation and neuroinflammatory changes, indicating retinal involvement beyond cortical damage 8. A key pathological feature of post-TBI visual impairment is retinal ganglion cell (RGC) damage, which contributes to progressive visual dysfunction and neurodegeneration 9. Several mechanisms may underlie this retinal pathology, including excitotoxicity, oxidative stress, blood‒brain barrier (BBB) disruption, and neuroinflammation 10. Damage to central visual processing regions, such as the lateral geniculate nucleus (LGN) and superior colliculus (SC), may further contribute to retrograde transsynaptic degeneration (RTD) of RGCs, driven by disrupted trophic support, axonal damage, and glial activation 11. Given the limited effective therapeutic strategies for TBI-induced RGC damage, novel neuroprotective interventions that promote RGC survival and functional recovery are urgently needed.

In animal models of RGC and optic nerve degeneration, mesenchymal stem cells (MSCs) or their secretome have shown robust neuroprotective effects, preserving RGC survival and visual function after optic nerve injury, glaucoma, and other retinopathies 12-14. More recently, systemic or locally delivered MSCs and MSC-derived secretome/extracellular vesicles have been reported to attenuate TBI-related damage, including blast-induced visual deficits and RGC loss, and to improve neurobehavioral outcomes in various TBI models 14. However, it remains unclear whether intravenously administered MSCs can simultaneously mitigate cortical and hippocampal injury and remote TBI-induced retinal degeneration in a repeated lateral fluid percussion model, and how this is linked to changes in retinal β-catenin and growth-factor signaling (vascular endothelial growth factor [VEGF]/VEGF receptor-2 [VEGFR2], hepatocyte growth factor [HGF]/c-Met, and insulin-like growth factor binding proteins [IGFBP-4, IGFBP-6]/insulin-like growth factor-1 receptor [IGF-1R]). Our study was designed to address this gap.

To address this gap, we utilized a dual-model approach combining both an *in vivo* repeated TBI rat model and an *in vitro* stretch injury (SI) model using R28 cells, a retinal precursor cell line, that shares biological features with RGCs. The* in vivo* model enables analysis of the systemic and remote effects of brain injury on both cortical and retinal structure and function. The* in vivo* model allows analysis of the systemic and remote effects of TBI on brain and retinal structure and function, whereas the *in vitro* SI model simulates direct mechanical strain-induced damage to RGCs, which may mimic aspects of axonal stretch and biomechanical forces experienced during head trauma 15. Although mechanical forces are not directly applied to the retina in the lateral fluid percussion (LFP) model, secondary injury mechanisms, including neuroinflammation, oxidative stress, blood-retinal barrier disruption, and retrograde transsynaptic degeneration, contribute to RGC loss. We acknowledge that the SI model does not capture this full spectrum of secondary responses. However, it provides a controlled and reproducible environment to isolate cell-intrinsic responses, such as mitochondrial dysfunction, oxidative stress, autophagy, and β-catenin downregulation, that occur in response to axonal strain. Therefore, the combined use of these two models allows us to distinguish between primary mechanical and secondary neurodegenerative effects and to evaluate the therapeutic effects of MSCs across both paradigms.

## Material and Methods

### Study design

**Figure 1:**
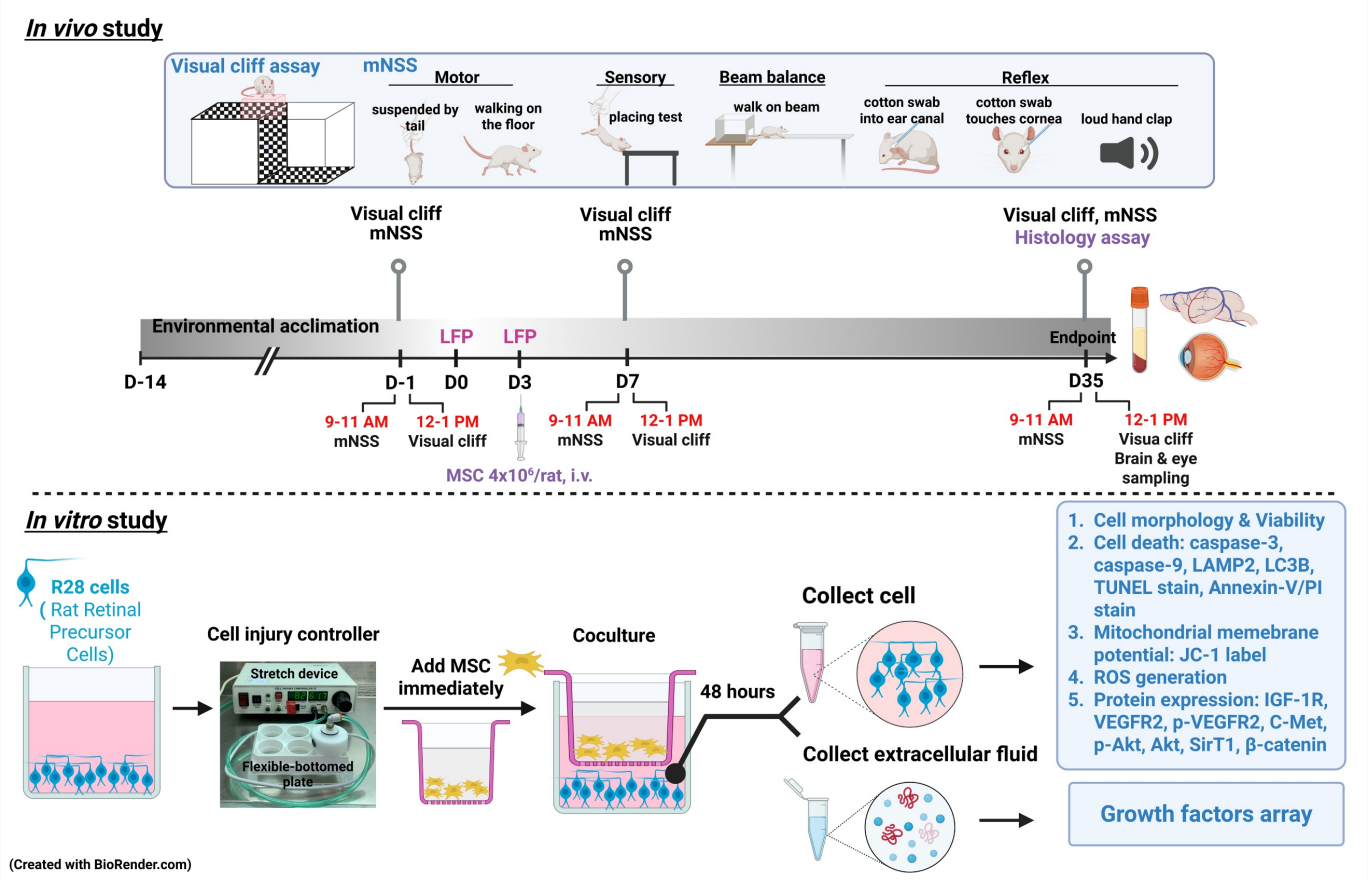
** Experimental design.** In *in vivo* study, male Wistar rats were divided into sham operation rats received vehicle (normal saline 1 ml/kg) treatment group (Sham+Veh), sham operation rats received MSC treatment (4 × 10^6^ cells/ml/kg) group (Sham+MSC), TBI rats received vehicle treatment (TBI+Veh) group, and TBI rats received with MSC treatment (4 × 10^6^ cells/ml/kg) group (TBI+MSC). Rats were acclimatized for two weeks and then subjected to visual and neurological function tests before surgery (-1 day or D-1). Rats received first LFP and second LFP on days 0 (D0) and 3 (D3), respectively. At day 35 (D35) of post-surgery, rats were euthanized after the last functional tests, and then the serum, brain, and eyes were collected for histological and biochemical examination. In the *in vitro* study, R28 cells were divided into non-stretch injury (SI) R28 cells cultured with control medium (C group), non-SI cells cocultured with MSC (C+MSC group), SI-R28 cells culture with control medium (SI group), and SI-R28 cells cocultured with MSC (SI+MSC group). The SI was performed by thrice 7.5-8.5 PSI impacts under the cell injury controller. The injured-R28 cells were cocultured with MSC immediately for 48 hours. Both the R28 cells and their extracellular fluid were separated, respectively. Cell viability was measured using an MTT assay. Cell apoptosis, mitochondria membrane potential, and ROS generation were analyzed using Annexin-V/PI, JC-1, and CM-H2DCFDA labeling combined with flow cytometry. The immunofluorescence staining was applied to verify the neuronal apoptosis. The R28 cell lysates were used to signal transduction protein marker identification. The human growth factors array analyzed the compositions of the growth factors in the extracellular fluid.

Both *in vivo* and *in vitro* experiments were conducted to explore the effects of MSCs on TBI or SI (**Fig. 1**). In the* in vivo* model, seventy-two male Wistar rats were divided into four groups: (1) sham-treated rats received 0.9% saline injection (sham+ Veh); (2) sham-treated rats received 4x10^6^ MSCs/ml/kg (sham+MSC); (3) rats with TBI received 0.9% saline injection (TBI+Veh); and (4) rats with TBI received 4x10^6^ MSCs/ml/kg (TBI+MSC) intravenously (i.v.) immediately after the second TBI. From day 7 (D7) to day 35 (D35) post-surgery, the following analyses were performed: (1) visual function and (2) neurobehavior assessment. After the final functional assessments on D35, animals were euthanized for serum and tissue collection. Brains and ocular tissues were harvested for 2,3,5-triphenyl tetrazolium chloride (TTC) and Luxol fast blue (LFB)/cresyl violet staining to evaluate brain contusion and white matter injury; hematoxylin and eosin (H&E) staining to assess brain and retinal histopathology; and immunofluorescence (IF) staining to analyze neuronal loss and gliosis in the brain, retinal ganglion cell apoptosis, and the expression of growth factors and their associated receptors. Ocular tissue and serum were used to evaluate the growth factor levels. In the* in vitro* model, R28 cells were used and assigned to the following four groups: (1) the control group (C); (2) control cells that were cocultured MSCs (C+ MSC); (3) the SI group (SI); and (4) SI-treated cells that were cocultured with MSCs (SI+ MSC). After 48 h of coculture, the following assays were conducted: (1) MTT assays for cell viability; (2) IF staining for cell apoptosis; (3) flow cytometry for cell death, mitochondrial membrane potential changes, and reactive oxygen species (ROS) generation; and (4) immunoblotting for cell death, growth factor arrays, and intracellular signaling. All the authors had access to the study data and reviewed and approved the final manuscript. All *in vivo* studies included eighteen animals per group. All *in vitro* studies involved at least 3 replicate experiments. All primary and secondary antibodies, as well as commercial assay kits used in this study, are detailed in **Table 1**.

### Animals

All animal procedures used in this study were approved by the Institutional Animal Care and Use Committee of Chi Mei Medical Center, Tainan, Taiwan (approved no. 110122319) and were conducted in accordance with the Association for Research in Vision and Ophthalmology Statement for the Use of Animals in Ophthalmic and Vision Research. The experimental design was conducted following the ARRIVE guidelines (Animals in Research: Reporting *In Vivo* Experiments) for the care and use of laboratory animals. We purchased 300-350 g Wistar rats from BioLASCO Taiwan Co., Ltd. (Taipei, Taiwan) and housed them at the Chi Mei Animal Center under a 12-h light/dark cycle and at 25 ± 1°C with a fixed humidity. We provided unlimited chow and fresh water. A statistical power analysis was performed to estimate the sample size. Given that all the parameters yielded large effect sizes, we could ensure that a total sample size of 72 rats (n = 18 per group, where n represents the number of rats) could detect neurobehavioral and histologically relevant differences with a power of 80%. The animals were randomly (https://www.randomizer.org/) allocated using a computer random group generator to one of 4 groups: the sham+Veh (n=18), sham+MSC (n=18), TBI+Veh (n=18), and TBI+MSC (n=18) groups. All the authors were aware of the group allocation at the different stages of the experiment. The rats were identified using a Stoelting™ Rat Ear Tag (Stoelting Co., Il, USA) for recognition and testing. Thirty-five days after surgery, twelve rats per group were euthanized, six for the contusion volume assay, and another six for immunohistological analyses.

### Induction of TBI

TBI was induced using the LFP model, which is a well-established method for replicating moderate TBI in rodents. Adult male Wistar rats were anesthetized with an intraperitoneal (i.p.) injection of Zoletil (50 mg/kg; Virbac, Nice, France), xylazine hydrochloride (2 mg/kg; Balanzine, Health-Tech Pharmaceutical Co., Taipei, Taiwan), or atropine sulfate (1 mg/kg; Tai Yu Chemical & Pharmaceutical Co. Ltd., Hsinchu, Taiwan). To maintain physiological body temperature and prevent hypothermia, we placed the rats on a warming pad (RightTemp® Temperature Monitor & Homeothermic Warming Control Module; Kent Scientific, Torrington, CT, USA) throughout the anesthesia period. Once anesthetized, the rats were positioned in a stereotaxic apparatus (David Kopf Instruments, Tujunga, CA, USA), and a 4.5-mm circular craniotomy was performed using an electric drill on the right lateral side of the skull at A/P -3 mm and M/L 4 mm with reference to the bregma suture 16. A modified luer-lock connector (trauma cannula), 2.6 mm inner diameter, was cemented into the craniotomy with dental acrylic and then connected to a fluid percussion device (VCU Biomedical Engineering, Richmond, VA, USA). TBI was induced by delivering a pressure of 1.5-1.6 atm on day 0 (D0) and a second injury at 1.5-1.6 atm on day 3 (D3). Sham animals underwent identical surgical procedures but did not receive fluid percussion injury. After the injury was induced, the scalp was closed with 4/0 sutures and treated with antibiotic ointment to prevent infection. For postoperative pain management, ketoprofen (5 mg/kg, twice daily, subcutaneous injection) was administered. The animals were kept on the warming pad during the immediate postanesthetic period to aid recovery and were monitored closely until they regained full consciousness. Daily wound care and monitoring were performed for one week to ensure proper recovery.

To confirm the severity of the induced TBI, we recorded apnea duration and righting reflex recovery time, which are widely used physiological indicators of injury severity 17, 18. Following LFP, the injured rats presented a mean duration of apnea of approximately 50 s, indicating a major physiological response to the injury. Additionally, we measured the righting reflex recovery time, which refers to the time required for the animal to return to an upright position after being placed on its back. The recorded righting reflex recovery time was 382 ± 90 s (mean ± SD). These values are consistent with the classification of moderate TBI in rodents and further validate the effectiveness of the injury model. Animals were excluded if they failed to regain spontaneous breathing within 90 s post-injury; had a righting reflex recovery time of less than 6.5 minutes or greater than 10 minutes, indicating deviation from the moderate TBI classification; or developed severe postsurgical complications, such as excessive bleeding, seizures, or infection. Additionally, any rats that exhibited substantial motor impairments before baseline neurobehavioral testing were excluded to ensure the accuracy of the functional assessments.

### Preparation of human MSCs

Human bone marrow-derived mesenchymal stem cells (hMSCs) were purchased from Sigma Millipore (#SCC034). For expansion, hMSCs were cultured in low-glucose Dulbecco's modified Eagle's medium (DMEM; #31600-034, Gibco) supplemented with 10% fetal bovine serum (FBS; #TMS-013-BKR, Sigma), 5 ng/mL basic fibroblast growth factor (bFGF; #GF003, Millipore), and 1% GlutaMax (#35050061, Gibco) at 37 °C in a humidified 5% CO₂ incubator. Cells between passages 3 and 6 were used for experiments. In our laboratory, hMSCs are routinely characterized by flow cytometry and tri-lineage differentiation, as described in our previous work 19. Briefly, these cells are positive for CD73, CD90, and CD105 and negative for CD34, CD45, and HLA-DR, and they exhibit osteogenic, chondrogenic, and adipogenic differentiation capacity. Representative characterization data have been published previously and are not repeated here. Before* in vivo* administration, hMSCs were detached, counted, and washed three times with sterile phosphate-buffered saline (PBS) to remove residual serum. Cells were then resuspended in serum-free PBS at a concentration of 4 × 10⁶ cells/mL/kg and injected via the tail vein on day 3 after surgery. Cell viability before injection was consistently >90% as assessed by trypan blue exclusion.

### Visual cliff test

The visual cliff model was used to test the depth perception and visual acuity of rats 8 on day 7 (D7) and day 35 (D35) after surgery. A transparent acrylic box was positioned on the edge of the laboratory bench. Half (length 50 cm × width 40 cm) of the base covered the bench, and the surface was lined with a checkerboard pattern (bench side or safe zone). The remaining surface (length 50 cm × width 25 cm; transparent side or cliff zone) was overhung approximately 60 cm above the floor and was lined with a similar checkerboard pattern to give the illusion of a “cliff”. An individual rat was placed on a small transparent acrylic box at the base at the edge of the cliff (on the bench side) and was allowed to survey its surroundings before dismounting the platform. Each rat was subjected to 10 trials. The apparatus was cleaned with 70% ethanol and water between each rat. The side chosen (checkerboard = safe or transparent = cliff) was recorded. The visual cliff behavior was averaged to obtain the number of trials in which the rat stepped down to the “cliff” side.

### Modified neurological severity score (mNSS)

The mNSS is a composite of motor (muscle status, abnormal movement), sensory (visual, tactile, and proprioceptive), and reflex tests. The score ranges from 0 to 18, and severity is defined as follows: 13-18, severe injury; 7-12, moderate injury; and 1-6, mild injury.

### Contusion volume assay by TTC staining

After the last neurobehavioral test, the rats were euthanized via a Zoletil overdose and transcardially perfused with 300 ml of 0.9% sodium chloride (w/v) with 0.11 ml of heparin (5000 IU/ml) in ice-cold saline. Rat brains were rapidly extracted, sliced into 1 mm coronal sections (Rat Brain Blocker, David Kopf Instruments, CA, USA), stained with 2% TTC (Sigma, Germany) for 30 min at 37 post-fixed dark, and post-fixed with formalin (4%). After photography with a digital camera, Image-Pro Plus (Media Cybernetics, MD, USA) software was used for contusion volume analysis, and the results were calculated as 1 mm (thickness of the slice) × (sum of the contusion area in all brain slices: mm^2^).

### Immunohistochemistry and immunofluorescence (IF) staining: *in vivo* model

The rats were euthanized via a Zoletil overdose and transcardial perfusion with 300 ml of 0.9% sodium chloride (w/v) with 0.11 ml of heparin (5000 IU/ml) followed by 4% paraformaldehyde. Eyes were fixed in Davidson solution for 24 h, and both eyes and brains were subsequently post-fixed in 4% paraformaldehyde (pH 7.0) for another 24 h at 4 °C before paraffin embedding. Brains and eyeballs were then embedded in paraffin, sectioned at 5 μm thickness at 50 μm intervals, and mounted on glass slides. Thirty sections per animal were prepared, with 10 each used for hematoxylin and eosin (H&E), Luxol fast blue (LFB)/cresyl violet, and immunofluorescence (IF) staining.

H&E staining was performed to evaluate both brain morphology and retinal structural integrity following TBI. After standard deparaffinization and rehydration, tissue sections were stained with hematoxylin and eosin and coverslipped. All images were captured using a Carl Zeiss upright light microscope equipped with a digital camera and Axioscope software (Zeiss, Jena, Germany). Brain coronal sections at the lesion core level were evaluated to determine histological injury severity. A semiquantitative scoring system was used to assess the cortex and hippocampus separately based on the degree of:

1. Neuronal loss or degeneration (e.g., pyknosis, karyolysis)

2. Tissue disorganization or cavitation

3. Vacuolization and edema

4. Inflammatory infiltration or gliosis

Each parameter was scored on a scale of 0 (none) to 3 (severe), yielding a total score of 0-12 for each region. Scores were averaged across three sections per region per rat (i.e., per cortex or hippocampus) for group comparisons.

For retinal evaluation, sections were taken at a standardized distance of 1500 μm from the optic nerve head. Measurements of:

1. Full retinal thickness

2. Ganglion cell complex (GCC) thickness

3. Ganglion cell nucleus (GCN) density

were obtained from four cross-sections per eye using Image-Pro Plus 6.0 software (Media Cybernetics, Rockville, MD, USA). These values were averaged per animal to assess retinal degeneration.

Histopathological assessment of H&E-stained brain and retinal sections was performed by a blinded investigator trained in neuroanatomy and ocular histology to evaluate cortical and hippocampal injury severity, as well as retinal structural integrity.

LFB/cresyl violet staining was performed to evaluate myelin integrity and white matter injury. Brain sections were incubated in 60 °C LFB for 1 h, differentiated in lithium carbonate and 70% ethanol, and counterstained with cresyl violet. Brain contusion volume was measured using Image-Pro Plus by outlining the spared ipsilateral and contralateral cortical regions.

Brain contusion volume (%) = (Contralateral area - Ipsilateral spared area) / Contralateral area × 100

Immunofluorescence staining was used to evaluate neuronal survival, gliosis, trophic factor signaling, and apoptosis in both brain and retinal tissues. Ten-micron coronal brain and retinal sections were blocked and incubated with primary antibodies against NeuN, GFAP, MAP2, c-Met, HGF, IGF-1R, IGFBP-4, IGFBP-6, VEGFR2, VEGF, or β-catenin, followed by appropriate Alexa Fluor-conjugated secondary antibodies. DAPI was used for nuclear counterstaining. For apoptosis detection, a TUNEL assay was performed prior to NeuN co-staining.

Images were captured at 200× or 400× magnification using a Carl Zeiss fluorescence microscope and analyzed using Zen software. Quantification was performed by a trained observer blinded to the experimental groups. For each section, NeuN+MAP2+DAPI, GFAP+DAPI, NeuN+TUNEL+DAPI, NeuN+β-catenin+DAPI, or NeuN co-labeled with c-Met+HGF, IGF-1R+IGFBP-4, IGF-1R+IGFBP-6, or VEGFR2+VEGF were quantified in six randomly selected 400× fields. This approach enabled a comprehensive analysis of cellular integrity, apoptosis, and neurotrophic signaling in both cortical and retinal regions.

### Enzyme-linked immunosorbent assay (ELISA) for serum and eye tissue growth factors

To quantify growth factor levels in serum and eye tissue, ELISA was performed for VEGF, HGF, IGFBP-4, and IGFBP-6 using commercial kits according to the manufacturers' instructions. For serum collection, whole blood was obtained from anesthetized rats via cardiac puncture, allowed to clot at room temperature for 30 minutes, and centrifuged at 3,000 × g for 15 minutes. Serum was collected and stored at -80 °C until analysis. For ocular tissue analysis, the entire eyeball was collected due to technical difficulties in isolating the retina alone. Ocular tissues were homogenized in ice-cold PBS (pH 7.4) containing protease inhibitors, then centrifuged at 12,000 × g for 15 minutes at 4 °C. Supernatants were collected and used for ELISA. Samples and standards were added to 96-well plates coated with capture antibodies (including VEGF, HGF, IGFBP4, and IGFBP6), incubated, and processed with biotin-labeled detection antibodies, streptavidin-HRP, and TMB substrate. Absorbance was read at 450 nm using a microplate reader (BioTek Instruments). Growth factor concentrations were calculated based on standard curves and normalized to sample volume or tissue protein concentration. All samples were performed in triplicate.

### R28 retinal neuron culture

Immortalized rat retinal precursor cells (R28) were purchased from Applied Biological Materials (Richmond, BC, Canada; #T0575). R28 cells were maintained in low-glucose DMEM (#31600-034, Gibco) supplemented with 10% bovine calf serum (#SAB-12133C, Sigma-Aldrich), 1% MEM non-essential amino acids (#11140-019, Gibco), 1% MEM vitamins (#11120-029, Gibco), and 1% penicillin/streptomycin (#15140122, Gibco) at 37 °C in a humidified 5% CO₂ incubator.

### Coculture of stretched and injured R28 cells and uninjured MSCs

To mimic traumatic mechanical stress, R28 cells were subjected to stretch injury (SI) using a Cell Injury Controller II (Custom Design and Fabrication, Virginia Commonwealth University). Briefly, R28 cells were seeded onto flexible-bottom culture plates (#BF-3001C, Flexcell) and, after reaching appropriate confluence, the culture medium was gradually switched to serum-free low-glucose DMEM (#11885084, Gibco) before SI in the injury groups. Mechanical injury was induced by applying a peak nitrogen pressure of 7.5-8.5 psi (approximately 0.51-0.57 atm) three times, producing a moderate SI with a center membrane deflection of ~10.5 mm.

For coculture experiments, hMSCs were seeded into cell culture inserts with a pore size of 0.4 µm (#PIHP03050, Millipore) and allowed to equilibrate in serum-free low-glucose DMEM. Immediately after SI, the culture medium of the SI and SI+MSC groups was replaced with serum-free low-glucose DMEM, and the MSC-containing inserts were placed on top of the R28 plates to establish a Transwell coculture system. Coculture was maintained for 48 h at 37 °C in 5% CO₂. Four experimental groups were studied: (1) Control (C): R28 cells on flexible-bottom plates without SI, maintained in standard R28 culture medium; (2) C+MSC: R28 cells without SI cocultured with MSCs in Transwell inserts, using standard R28 culture medium; (3) SI: R28 cells subjected to SI and then maintained in serum-free low-glucose DMEM; (4) SI+MSC: R28 cells subjected to SI and then cocultured with MSCs in serum-free low-glucose DMEM.

After 48 h, R28 cell lysates were collected for assays of cell viability, apoptosis, mitochondrial membrane potential, reactive oxygen species (ROS) production, autophagy markers, and protein signaling, and conditioned media were harvested for growth factor array and ELISA analysis.

### MTT cell viability assays

The viability of R28 cells was determined via the use of 3-(4,5-dimethyl-thiazol-2-yl)-2,5-diphenyltetrazolium bromide (MTT; #298931, Serva Electrophoresis GmbH, Heidelberg, Germany). After coculture, the R28 cells were incubated with MTT solution for 3 h at 37°C, and the optical density of each sample was measured at 540 nm with a spectrophotometer (MultiSkan GO, Thermo Fisher Scientific, Waltham, MS, USA). The values are presented as percentages relative to the control group (defined as 100% survival).

### IF staining: *in vitro* model

The cells were fixed with 4% paraformaldehyde for 10 min and permeabilized with 0.3% Triton X-100 in PBS for 5 min at room temperature. The cells were blocked from nonspecific binding with background reducing buffer (#TA00B3-100, BioTnA) for 1 h at room temperature, and TdT labeling reagent was added for the TUNEL assay (Cat#TAAP01F-100, BioTnA Biotech, Kaohsiung, Taiwan). After being washed with PBS three times, the cells were incubated with MAP-2 and β3-tubulin antibodies at 4 °C overnight. After being washed with PBS, the cells were incubated with appropriate secondary antibodies, then stained with rhodamine-phalloidin to label F-actin and DAPI for nuclear counterstaining. Digital images were captured with a 40x objective (numerical aperture [NA] 0.75) and a 100x oil immersion objective (NA 1.4) by an upright fluorescence microscope system (Carl Zeiss Microscopy GmbH, Jena, Germany) with Zen Software (Carl Zeiss). MAP2-, β3-tubulin-, and TUNEL-positive cells were counted. The data are presented as the number of TUNEL-expressing neurons in four fields per coverslip and six coverslips per experimental condition.

### Immunoblotting

R28 cells were washed with PBS and dissociated with TrypLE^TM^ Express (#12604‒021, Gibco). Then, the cells were lysed on ice in 1× RIPA buffer (50 mM Tris pH 7.5, 150 mM NaCl, 0.1% Nonidet P-40, 0.2% deoxycholate, and 100 mM EDTA) freshly supplemented with protease and phosphatase inhibitor mixtures (Cell Signaling Technology, Beverly, MA, USA). The lysates were homogenized, and the protein supernatants were collected for further protein assays. Protein concentrations were quantified with a protein assay dye (#5000006; Bio-Rad, Hercules, CA, USA). Equal amounts of total protein were resolved by sodium dodecyl sulfate‒polyacrylamide gel electrophoresis (SDS‒PAGE), transferred to PVDF membranes, and blocked in nonfat milk in PBS containing 0.05% Tween-20 (Sigma). The membranes were immunoblotted with caspase-3, caspase-9, LAMP2, LC3B-I & II, IGF-1R, VEGFR2, p-VEGFR2, c-Met, Akt, p-Akt, SirT1, b-catenin, and b-actin antibodies at 4 °C overnight. After washing, the membranes were incubated at room temperature for 2 h with horseradish peroxidase-conjugated anti-rabbit/mouse IgG secondary antibodies at a 1:5,000~10,000 dilution (Cell Signaling Technology). Immunoreactive bands were visualized with enhanced chemiluminescence solution (#NEL10400, PerkinElmer, Waltham, MA, USA), and images were captured with X-ray film. The band intensities were measured using ImageMaster software (TotalLab, Amersham Biosciences, NJ, USA) and normalized to the intensities of the respective housekeeping protein bands.

### Flow cytometry with Annexin-V/propidium iodide (PI) staining

According to the manufacturer's instructions, apoptotic cells were detected using an Annexin V-FITC/PI kit (BD Biosciences, San Jose, CA, USA). In brief, the cells were collected and resuspended in binding buffer containing Annexin V-FITC and PI. The cells were incubated for 20 min at room temperature in the dark and then examined with Novocyte flow cytometry (ACEA Biosciences, CA, USA) (excitation: 488 nm and emission: 525 nm and 617 nm for FITC and PI, respectively). Data analysis was performed, and the relative number of Annexin V/PI-stained cells in the population was quantified via Novoexpress software.

### Analysis of the mitochondrial membrane potential

Changes in the mitochondrial membrane potential were determined with the fluorescent dye JC-1 (BD MitoScreen Kit, San Jose, CA, USA). In brief, the cells were stained with JC-1 and incubated in the dark at 37 °C for 15 min. The samples were subsequently added to the staining buffer and analyzed on a Novocyte flow cytometer. Relative changes in mitochondrial polarization were quantified by measuring the redshifted JC-1 aggregates in the FL-2 channel and the greenshifted monomers in the FL-1 channel. The results are expressed as percentages.

### ROS measurement

After treatment, the cells were harvested and stained with 10 µM CM-H_2_DCFDA (a ROS indicator, #C6827; Thermo Fisher Scientific, Waltham, MA, USA) or Total ROS/Superoxide detection kit (ENZO Life Science, NY, USA) for 30 min at 37 °C. Following incubation, the cells were washed and then resuspended in PBS. ROS production was measured via a Novocyte flow cytometer at excitation/emission wavelengths of 488/528 nm.

### Human growth factor antibody array

A human growth factor antibody array (ab134002, Abcam, Cambridge, MA) containing 41 targets was used to determine the expression of proteins secreted from the conditioned medium. The array membrane was blocked at room temperature for 30 min with a blocking buffer and then incubated with a conditioned medium sample for 2 h at room temperature. After washing, the biotin-conjugated anti-cytokine agent was added, and the mixture was incubated with the membrane overnight at 4 °C, washed, and then hybridized with HRP-conjugated streptavidin overnight at 4°C. The bound antibodies were subsequently visualized via a mixture of buffers C and D and autoradiography. The relative intensity of each signal dot was determined using ImageMater software (TotalLab, Amersham Biosciences, NJ, USA).

### Statistical analysis

Statistical analyses were performed using GraphPad Prism 7.01 (GraphPad Software, Inc., CA, USA). Parameters such as brain contusion, retina thickness, and IF staining data with a non-normal distribution were analyzed via the Kruskal‒Wallis test and Dunn's post hoc test. For neurobehavioral and visual function analyses, a two-way repeated-measures analysis of variance (ANOVA) with Tukey's multiple comparisons test was conducted to assess group and time effects following surgery. One-way ANOVA with Tukey's post hoc test was used to analyze differences in enzyme-linked immunosorbent assay (ELISA), flow cytometry, growth factor array, and Western blot data. All the data are expressed as the means ± standard deviations (SDs). P-values < 0.05 were considered statistically significant.

## Results

### MSC therapy attenuated TBI-induced visual dysfunction, neurological deficits, brain injury, and neuronal loss in rats

**Figure 2:**
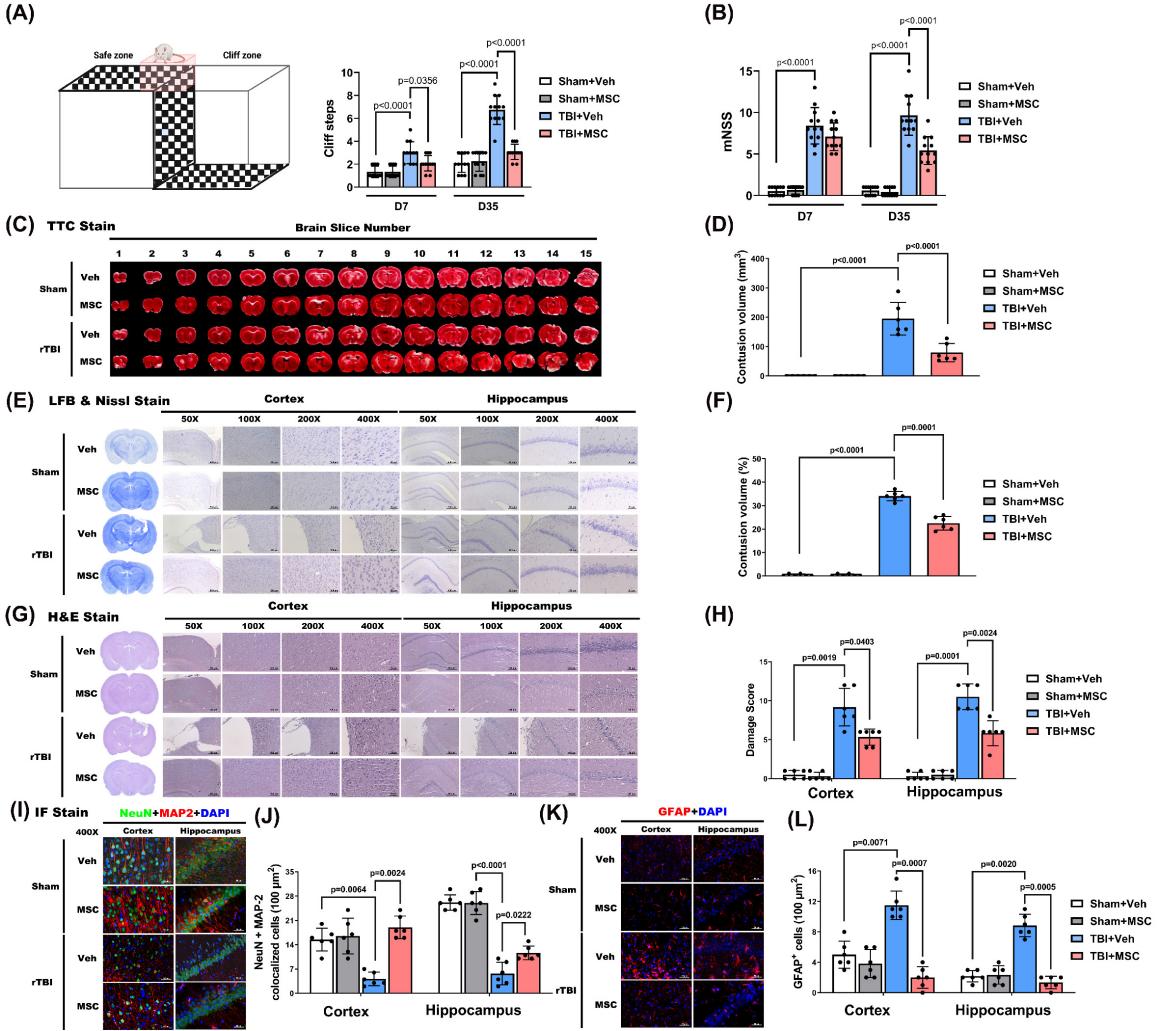
** MSC therapy ameliorated traumatic brain injury-induced visual dysfunction, neurological disorder, and retinal injury in rats. (A)** Schematic of the visual cliff apparatus and quantification of cliff steps on days 7 (D7) and 35 (D35) post-injury. TBI+Veh rats showed significantly more cliff steps compared to Sham+Veh rats, indicating impaired depth perception, which was attenuated by MSC therapy. **(B)** Modified Neurological Severity Score (mNSS) on D7 and D35. TBI+Veh rats exhibited significantly higher scores than Sham+Veh rats, reflecting neurological impairment. MSC treatment significantly improved mNSS in TBI rats. **(C)** Representative TTC-stained brain slices across 15 coronal sections. **(D)** Quantification of contusion volume in mm³. **(E)** Representative Nissl staining and **(F)** percentage of total brain volume. **(G)** H&E staining of the cortex and hippocampus at different magnifications (50×- 400×) and **(H)** Semiquantitative damage scoring of the cortex and hippocampus. **(I)** Representative immunofluorescence staining for neuronal marker NeuN (green), astrocytic marker GFAP (red), dendritic marker MAP2 (red), and DAPI (blue) in the cortex and hippocampus. Merged images show colocalization. **(J)** Quantification of NeuN+MAP2 colocalized cells per 100 μm² and GFAP+ cells per 100 µm^2^ in cortex and hippocampus. Data were obtained from 12 (for functional assay) or 6 (for histological assay) animals in each group and expressed as mean±SD.

To assess the impact of MSC therapy on TBI-induced brain contusion, we first evaluated visual function via the visual cliff test at D7 and D35 post-TBI. At D7 and D35, the TBI+Veh group exhibited significantly greater cliff-step errors than the sham+Veh group did (p < 0.0001, **Fig. 2A**), indicating delayed onset of visual impairment. Compared with those in the TBI+Veh group, MSC therapy significantly reduced cliff-step errors at D7 and D35 (p = 0.0356 and p < 0.0001, **Fig. 2A**), supporting a role for MSC therapy in visual function recovery.

To assess TBI-induced neurological function impairment, we determined the mNSS at D7 and D35 post-TBI. The TBI+Veh rats showed significantly higher mNSS scores than the sham+Veh rats at both time points (p < 0.0001; **Fig. 2B**), confirming persistent neurological impairment. However, at D35 post-TBI, not D7, MSC administration, compared with the TBI+Veh group, significantly reduced mNSS (p < 0.0001; **Fig. 2B**), suggesting functional recovery.

We next evaluated structural brain damage using TTC and Nissl stainin. As shown in **Fig. 2C and E**, the TBI+Veh group presented a significantly greater contusion volume than did the sham+Veh group on day 35 post-surgery (p < 0.0001, **Fig. 2D and F**). MSC administration significantly reduced brain contusion volume compared with the TBI+Veh group (p < 0.0001 and p = 0.0001, **Fig. 2D and F**), indicating a neuroprotective effect.

H&E staining further revealed that TBI caused extensive cortical and hippocampal tissue disorganization and neuronal loss (**Fig. 2G**), which were markedly ameliorated in MSC-treated rats. Semiquantitative histological scoring confirmed significant reductions in damage severity following MSC therapy (p = 0.0403 in cortex, p = 0.0024 in hippocampus, **Fig. 2H**).

In parallel, immunofluorescence staining showed that TBI induced neuronal loss and astrogliosis, as evidenced by decreased NeuN+MAP2 co-labeled neurons (**Fig. 2I** and** 2J**) and increased GFAP^+^ astrocytes in the cortex and hippocampus (**Fig. 2K** and** 2L**). Quantification demonstrated that MSC treatment significantly increased NeuN+MAP2 co-labeled cell density (p = 0.0024 in cortex, p = 0.0222 in hippocampus, **Fig. 2J**) while reducing the number of GFAP^+^ cells in both regions (p = 0.0007 in cortex, p = 0.0005 in hippocampus, **Fig. 2L**), suggesting preserved neuronal integrity and attenuated gliosis.

### MSCs attenuated TBI-induced retinal damage, neuronal apoptosis, and β-catenin dysregulation

**Figure 3:**
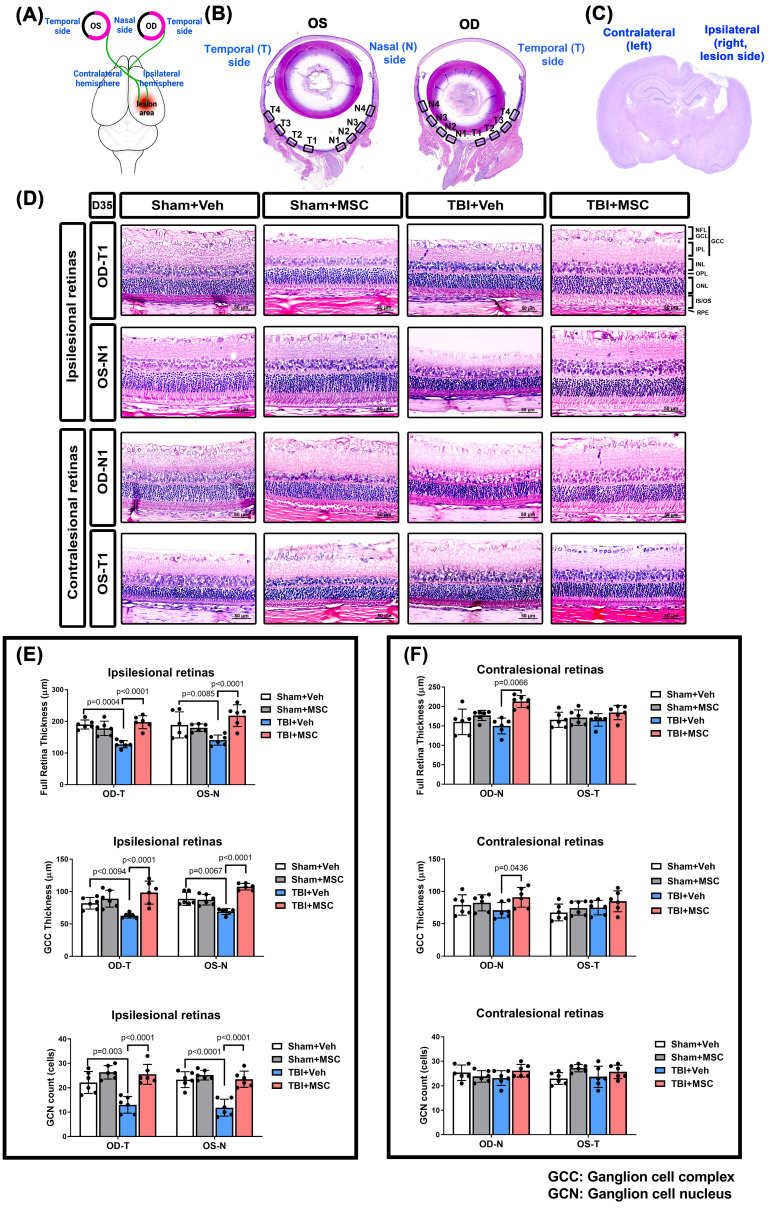
** MSC therapy reversed the TBI-induced reduced full retina, reduced GCC thickness, and reduced GCN counts in both OD and OS of the ipsilesional and contralesional retinas in rats. (A)** Schematic representation of the damaged brain region and correspondence visual pathway in the rat.** (B)** Representative HE-staining image of panoramic eye globe slice of Sham+Veh rat. In each eye, four nasal side areas (N1-N4) and four temporal side areas (T1-T4) were selected to quantify. **(C)** The HE-stained brain section denotes the LFP-induced brain lesion side (ipsilateral) and contralateral side. **(D)** Representative light microscopic images of H&E staining for a Sham+Veh, a Sham+MSC, a TBI+Veh, and a TBI+MSC rat at D35 after surgery. All images were obtained at 400× magnification. Scale bar = 50 µm. The full retinal and GCC thickness and ganglion cell nucleus (GCN) number in N1-N4 and T1-T4 of oculus dexter (OD, right eye) and oculus sinister (OS, left eye) in the **(E)** ipsilesional retinas and **(F)** contralesional retinas were quantified, respectively. Data were obtained from 6 animals in each group and expressed as mean±SD. NFL/GCL, nerve fiber layer, and ganglion cell layer; GCC, ganglion cell complex; IPL, inner plexiform layer; INL, inner nuclear layer; OPL, outer plexiform layer; ONL, outer nuclear layer; IS/OS, photoreceptor inner segment/outer segment; RPE, retinal pigment epithelium.

To investigate the retinal structural changes following TBI, we performed H&E staining of the right (Oculus Dexter, OD) and left (Oculus Sinister, OS) eyes (**Fig. 3**). Histological analysis (**Fig. 3A** shows the damaged brain region and corresponding visual pathway, and **Fig. 3B and 3D** show the panoramic and 400× magnification of the right eye [OD] and left eye [OS] by H&E staining; **Fig. 3C** shows the coronal section of the brain by HE staining) revealed that the TBI+Veh rats presented significantly thinner ganglion cell complexes (GCCs) and full retinal layers than the sham+Veh rats did in both the OD temporal (OD-T) and OS nasal (OS-N) regions (**Fig. 3E** and **3F**). Additionally, the TBI+Veh rats presented a significant reduction in ganglion cell nucleus (GCN) density in both the OD-T and OS-N regions (**Fig. 3E** and **3F**). In contrast, the MSC-treated rats presented preserved full retinal and GCC thickness and GCN density, indicating a protective effect on retinal integrity.

**Figure 4:**
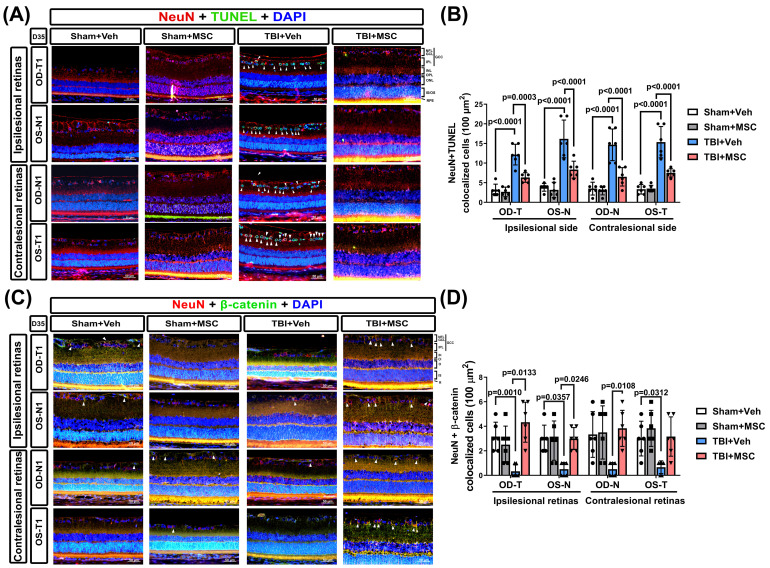
** MSC therapy reversed the TBI-induced neuronal apoptosis and loss of β-catenin-containing neurons in GCC of both eyes. (A)** Representative immunofluorescence images from the right and left retinas of rats in the Sham+Veh, Sham+MSC, TBI+Veh, and TBI+MSC groups. Red, green, and blue denote NeuN (neuronal marker), TUNEL (apoptosis marker), and DAPI (nuclear marker), respectively. The TUNEL signal observed in the Sham+MSC group represents background staining and does not indicate apoptotic neurons (see supplementary Figure S1 single-channel images for further validation). **(B)** Quantification of TUNEL-positive neurons in the GCC demonstrates that TBI significantly increases neuronal apoptosis (p < 0.0001), whereas MSC therapy reduces apoptosis (p < 0.0001 vs. TBI+Veh). **(C)** Representative immunofluorescence images of β-catenin-containing neurons in the retina. β-Catenin is shown in green, and NeuN in red. Arrows denote β-catenin-positive neurons in the retinal ganglion cell layer. **(D)** Quantification of NeuN+β-catenin double-positive cells using Image-Pro Plus. All images were obtained at 400× magnification. Scale bar = 50 µm. Data were obtained from six animals per group and are presented as mean ± SD.

To examine TBI-induced neuronal apoptosis in the retina, we performed NeuN/TUNEL IF staining (**Fig. 4A**). Single-channel fluorescence images of NeuN, TUNEL, and DAPI are provided in **Supplementary Figure S1** to ensure clear visualization of each marker. Compared with the sham+Veh rats, the TBI+Veh rats presented significantly greater numbers of apoptotic neurons (NeuN/TUNEL colocalized cells) in the GCC of both eyes (p < 0.0001, **Fig. 4B**). MSC administration significantly reduced neuronal apoptosis (p = 0.0003 ~ p < 0.0001, **Fig. 4B**), suggesting that MSC therapy protects retinal neurons from TBI-induced cell death.

To explore the role of β-catenin signaling in TBI-induced retinal damage, we quantified β-catenin expression in the ipsilesional and contralesional retinas via IF staining (**Fig. 4C**). The TBI+Veh rats presented a significantly lower β-catenin signal intensity than the sham+Veh rats did (p = 0.001 for OD-T; p = 0.0357 for OS-N; p = 0.0312 for OS-T; **Fig. 4D**). Notably, the MSC-treated rats presented significantly greater β-catenin expression than did the TBI+Veh rats (p = 0.0133 for OD-T; p = 0.0246 for OS-N; p = 0.0108 for OD-N; **Fig. 4D**), indicating that MSC therapy helps restore β-catenin signaling in the retina.

### MSC therapy restores viability and reduces apoptosis in SI-injured R28 cells

**Figure 5:**
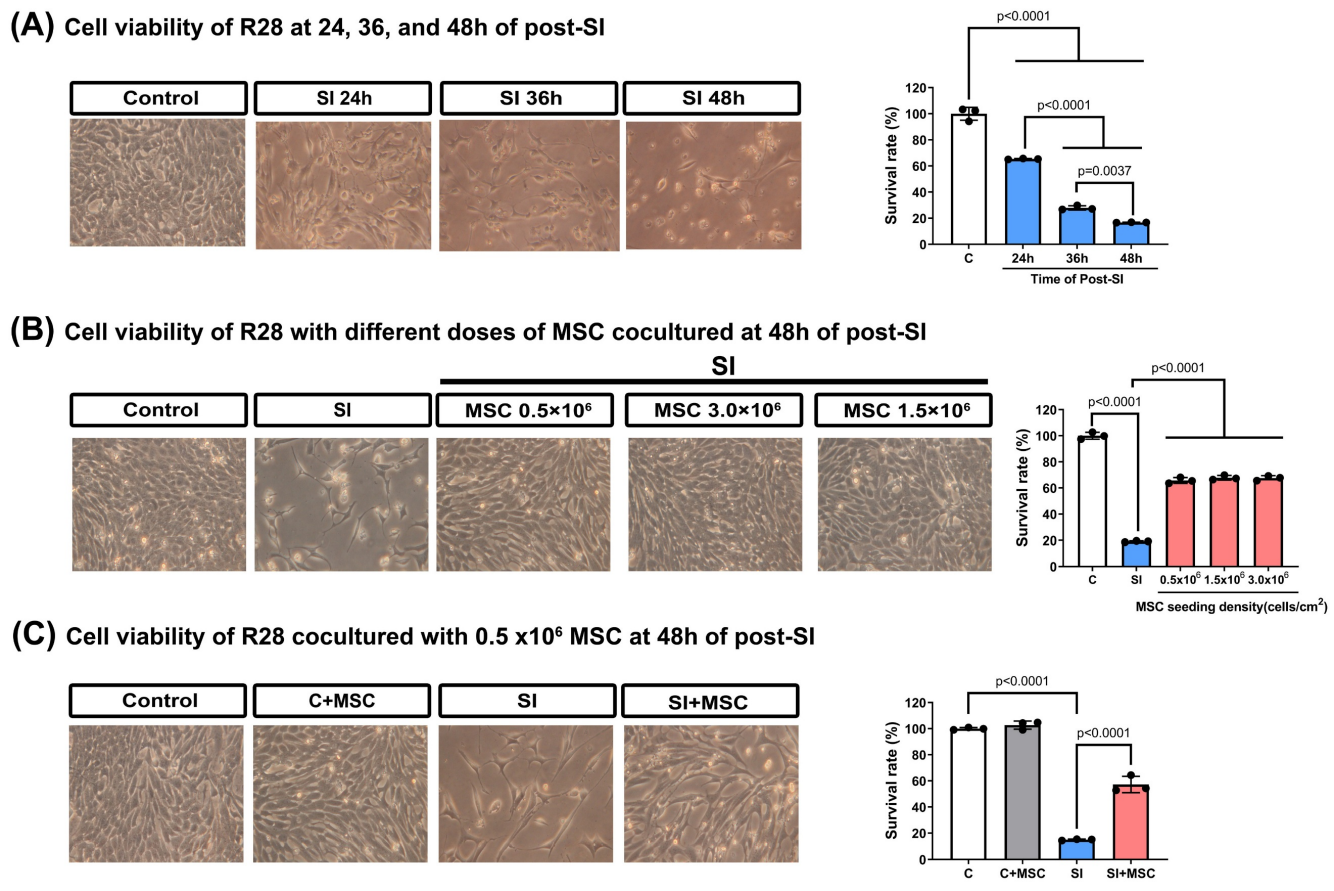
**MSC therapy reversed the stretch injury-induced reduced viability in R28 cells.** (A) Representative photographs of R28 cell morphological changes under stretch injury. The R28 cell survival rate was reduced in a time-dependent manner after stretch injury (SI). (B) Different doses (from 0.5 × 10^6^ to 3.0 × 10^6^) of MSC coculture 48 hours could attenuate the decrease of R28 cell viability caused by SI. (C) At 48 h of post-SI, compared to the control group, the SI group significantly reduced the R28 survival rate, which could be attenuated by 0.5 × 10^6^ MSC coculture (SI+MSC group). Represented data are from an individual experiment with three biological replicates. Data are mean ± SD.

To evaluate the effects of mechanical trauma on retinal cells, we subjected R28 (retina precursor cells) to SI and monitored their viability over time. SI resulted in a significant, time-dependent reduction in cell survival, with the most pronounced decline observed at 48 hours post-injury (**Fig. 5A**). Coculturing R28 cells with MSCs significantly increased the survival rates (p < 0.0001, **Fig. 5B**), with an optimal MSC dose of 0.5 × 10⁶ cells used for further experiments (**Fig. 5C**).

**Figure 6:**
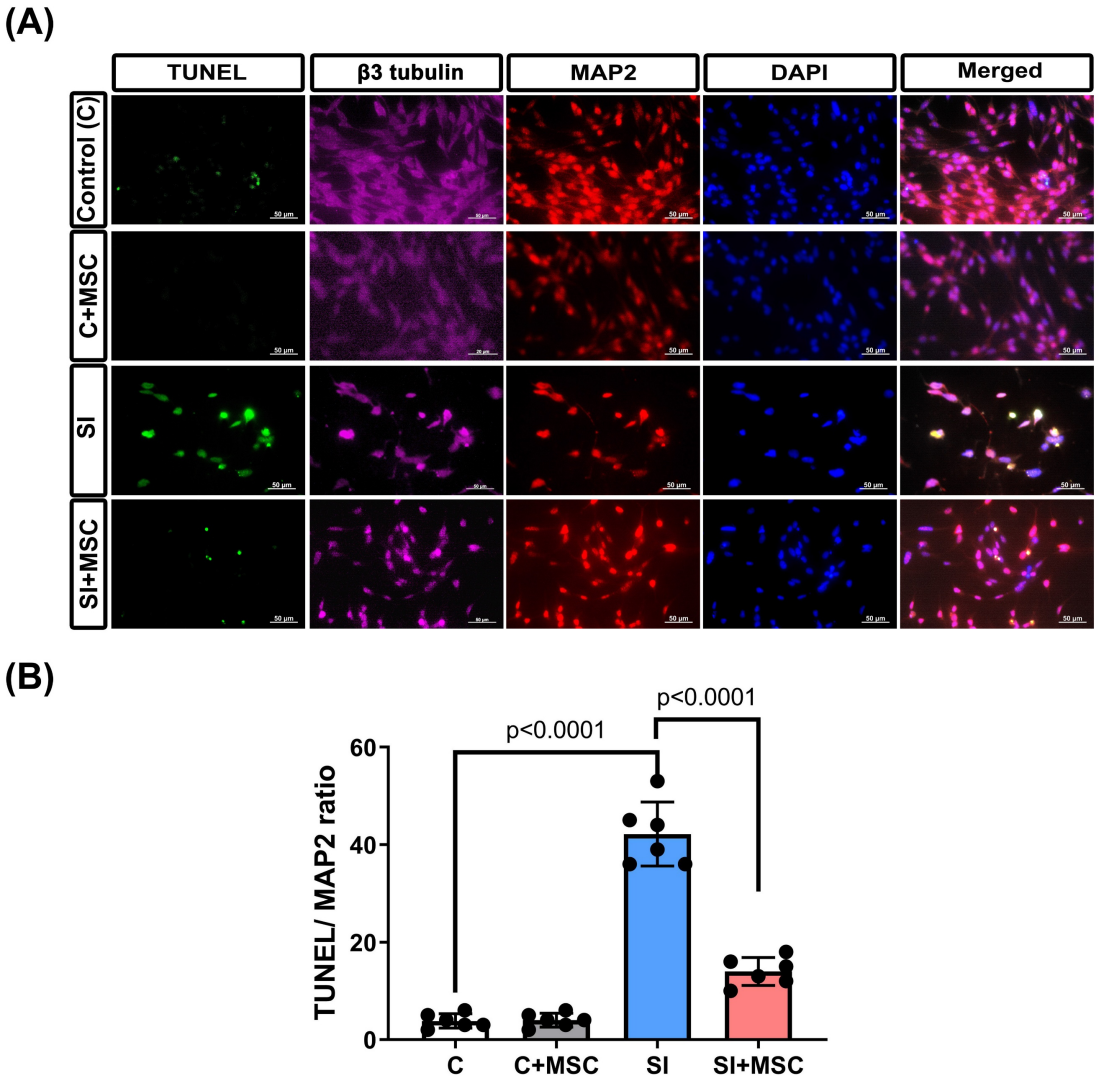
** MSC therapy reversed the stretch injured-induced neuronal apoptosis in R28 cells.** (A) Immunofluorescent staining of class III β-tubulin (β3 tubulin, pink), MAP2 (red), and TUNEL (green) in different groups of R28 cells. Scale bar = 50 µm. The cell nuclei in all panels were stained with DAPI and shown in blue. (B) Bars represent an individual experiment with three biological replicates and are present as mean ± SD.

To assess neuronal damage, immunofluorescence staining for TUNEL, MAP2, and βIII-tubulin was performed (**Fig. 6A**). MAP2 was used to label dendrites and evaluate neuronal integrity 20, whereas βIII-tubulin staining (not quantified) was included to provide a qualitative assessment of axonal structure 21. Compared with the control R28 cells (C group), the SI-injured R28 cells (SI group) presented significantly higher apoptosis rates, as indicated by increased TUNEL/MAP2 colocalization (p < 0.0001, **Fig. 6B**). MSC coculture (SI+MSC group) significantly reduced SI-induced apoptosis (p < 0.0001, **Fig. 6B**), supporting its protective effect on neuronal survival.

**Figure 7:**
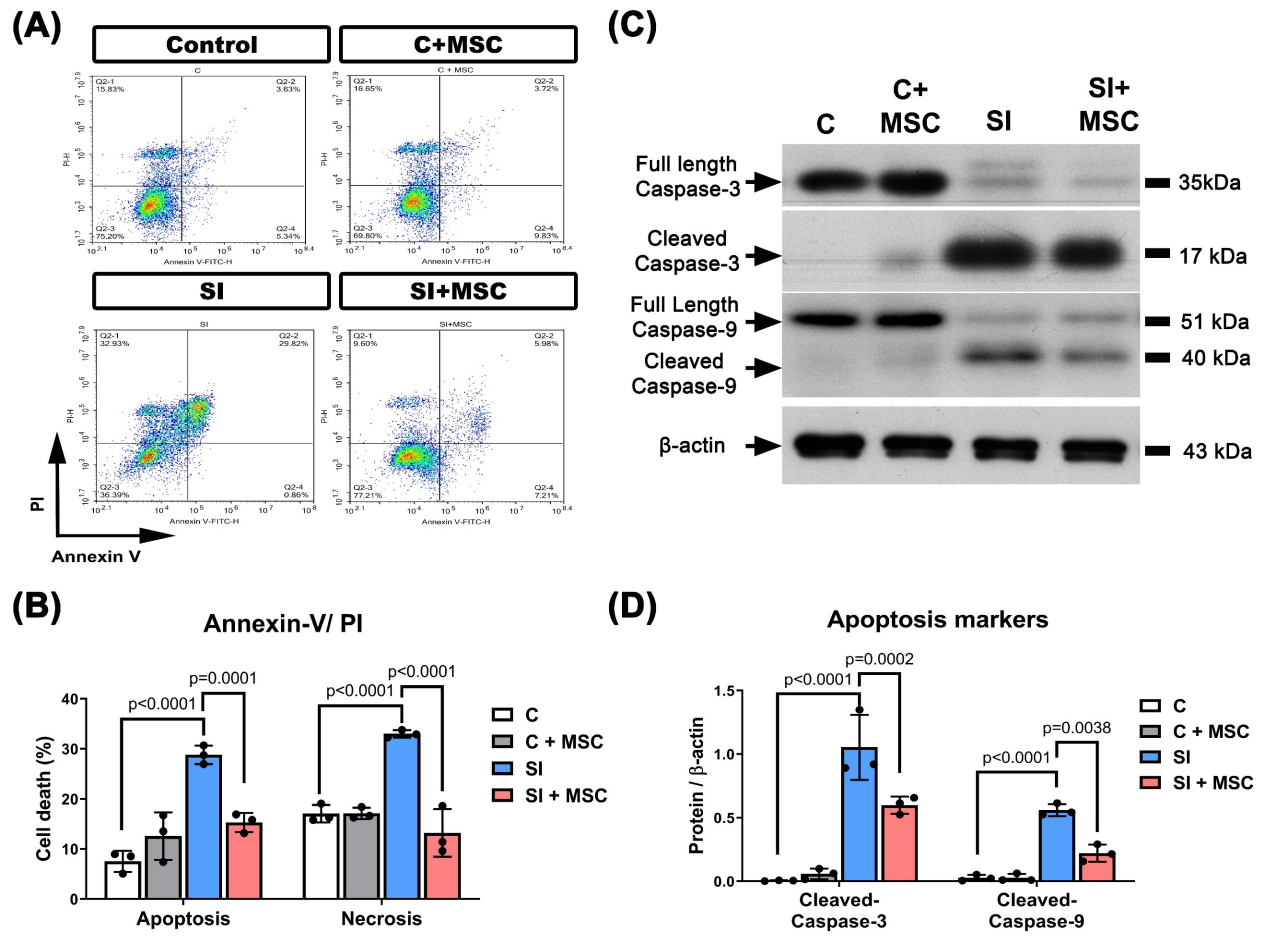
** MSC coculture reduced the R28 apoptosis caused by SI.** (A) Representative flow cytometer images of R28 cells with different treatments of MSC and SI. The y-axis quantifies the number of cells stained with propidium iodide (PI), and the x-axis quantifies the number of cells stained with Annexin V. (B) The percentage of apoptotic cells represents cells that are Annexin V positive, and both PI and Annexin V positive. The percentage of necrotic cells represents cells that are PI-positive. SI-induced R28 apoptosis and necrosis could be reduced significantly by MSC coculture. (C) The cleaved caspase-3 and caspase-9 levels were detected by Western blot analysis after the indicated treatments. β-actin was used as an endogenous control. (D) The relative cleaved caspase-3 and caspase-9 levels were indicated as a normalization of the cleaved caspase-3 or caspase-9/β-actin ratio in each sample. The data are presented as the mean ± SD of three independent experiments. For original images of the blots, please see the Supplementary Data file.

Consistent with these findings, flow cytometric analysis revealed significantly higher proportions of apoptotic and necrotic R28 cells in the SI group compared to controls (p < 0.0001 and p < 0.0001, respectively;** Fig. 7A** and **7B**), indicating that mechanical trauma induces substantial cell death. Western blot analysis further confirmed that SI-induced apoptosis was associated with increased expression of cleaved caspase-3 and caspase-9 (p < 0.0001, **Fig. 7C** and **7D**), which were significantly attenuated by MSC coculture (p = 0.0002 in cleaved-caspase-3 and p = 0.0038 in cleaved-caspase-9, **Fig. 7C** and **7D**; see **Supplementary Figure S2** for original blots). These results collectively demonstrate that MSCs reduce mechanical injury-induced cell death in retinal precursor cells through both structural preservation and inhibition of apoptosis-related signaling.

### MSC therapy alleviates mitochondrial dysfunction, oxidative stress, and excessive autophagy

**Figure 8:**
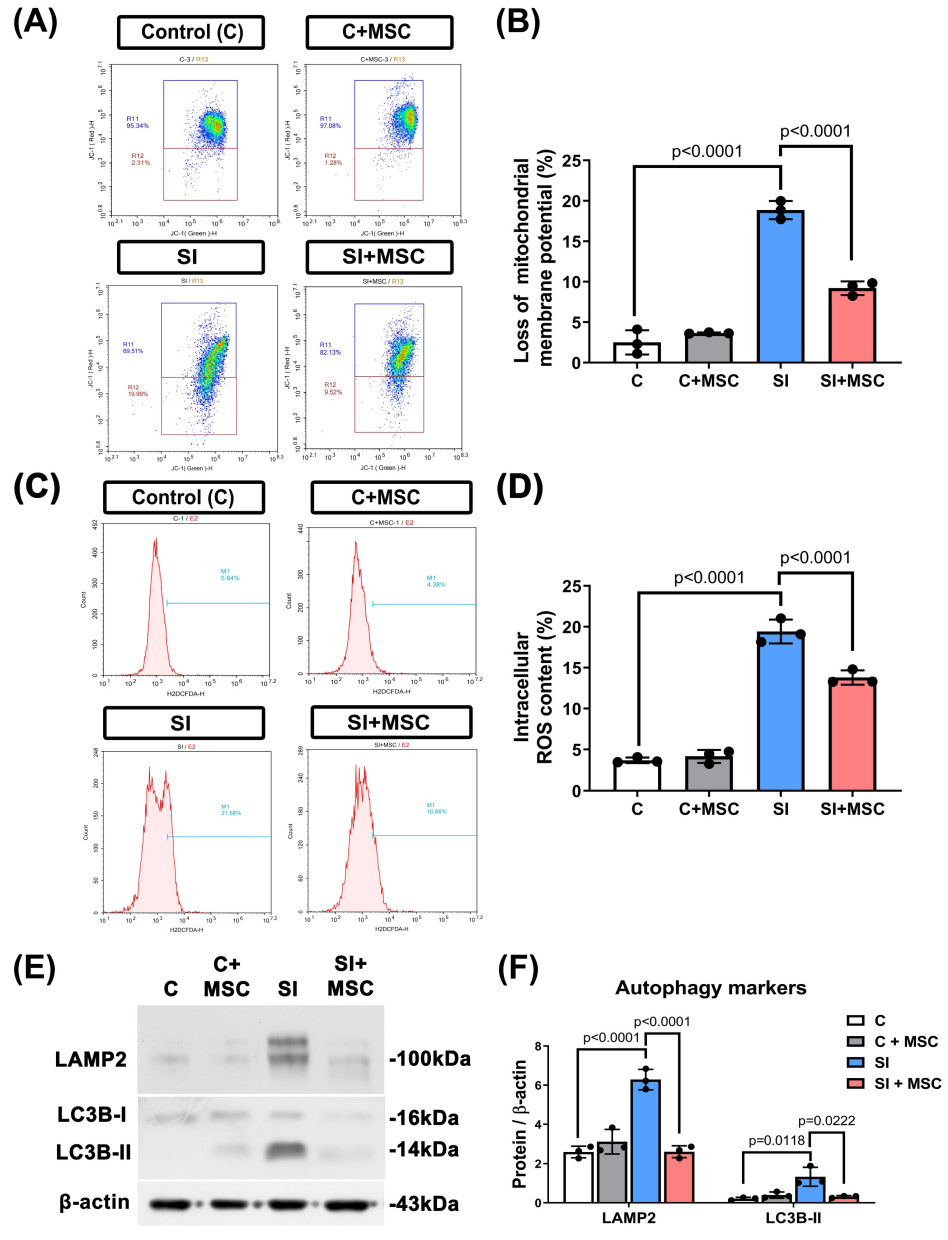
** MSC therapy reversed the SI-induced increased levels of autophagy markers, increased loss of mitochondrial membrane potential, and increased intracellular ROS contents in R28 cells. (A)** A dot plot displays the fluorescence density of J-aggregates (y-axis) against JC-1 monomers (x-axis). **(B)** Quantitative analysis of the loss of MMP by the red and green fluorescence ratio. **(C)** DCFH-DA probe with flow cytometry was used to detect the intracellular ROS levels in R28 cells. **(D)** The intracellular content of ROS was significantly increased by SI, which could be alleviated by MSC coculture. **(E)** Western blot analyses show the expression of several autophagy protein markers (LAMP2 and LC3B-II). **(F)** Bars are represented as the mean ± SD of three independent experiments. Represented data are from an individual experiment with three biological replicates. Data are mean ± SD. For original images of the blots, please see the Supplementary Data file.

To further explore the cellular mechanisms of SI-induced injury, we assessed mitochondrial membrane potential, autophagy markers, and intracellular reactive oxygen species (ROS) levels in R28 cells. SI caused a significant loss of mitochondrial membrane potential (p < 0.0001, **Fig. 8A** and **8B**), as well as increased intracellular ROS accumulation (p < 0.0001, **Fig. 8C** and **8D**). Additionally, autophagy was markedly elevated in SI-injured cells, as indicated by increased expression of lysosome-associated membrane protein 2 (LAMP2; p < 0.0001) and microtubule-associated protein 1A/1B-light chain 3B-II (LC3B-II; p = 0.0118) (**Fig. 8E** and **8F**). For original images of the blots, please see **Supplementary Figure S3**.

MSC coculture significantly reversed these effects: it preserved mitochondrial membrane potential (p < 0.0001), reduced ROS levels (p < 0.0001), and downregulated LAMP2 and LC3B-II expression (p < 0.0001 and p = 0.0222, respectively). These findings suggest that MSCs alleviate mitochondrial distress and oxidative stress while modulating autophagy to support neuronal homeostasis (**Fig. 8**).

### MSCs increased the expression of growth factors and receptors in stretch-injured R28 cells and rats with TBI

**Figure 9:**
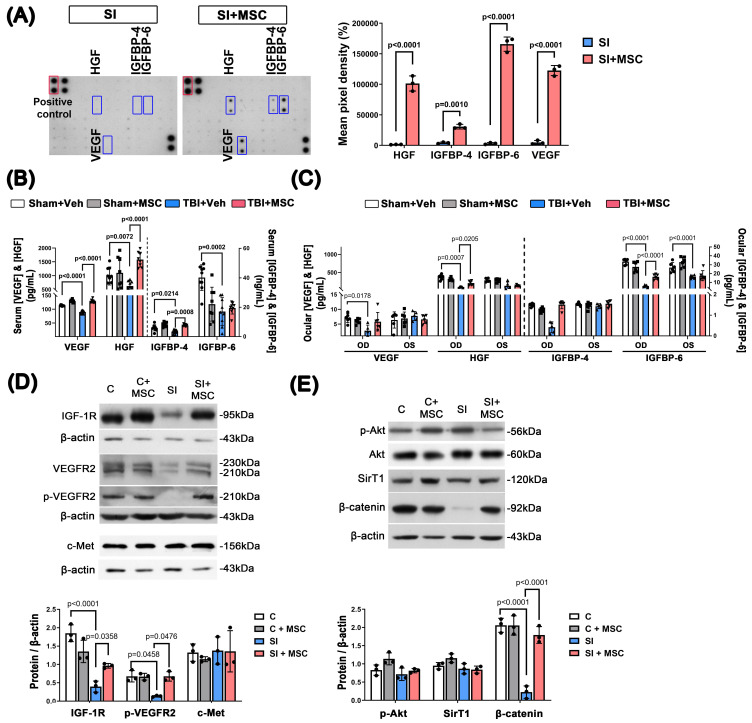
** MSC therapy enhances growth factor expression and receptor signaling in SI-injured R28 cells and TBI rats. (A)** Representative growth factor antibody array images from conditioned media of R28 cells subjected to stretch injury (SI), with or without MSC coculture. Increased secretion of HGF, IGFBP-4, IGFBP-6, and VEGF was observed in the SI+MSC group. Positive controls are marked in red boxes; significantly regulated proteins are highlighted in blue. Quantitative analysis of mean pixel density is shown. **(B)** ELISA quantification of serum VEGF, HGF, IGFBP-4, and IGFBP-6 levels in TBI rats. TBI reduced systemic levels of these growth factors, while MSC treatment significantly restored them (except IGFBP-6). **(C)** ELISA quantification of VEGF, HGF, IGFBP-4, and IGFBP-6 in whole eyeball lysates (OD and OS). Due to the technical difficulty in isolating the retina alone, entire ocular globes were homogenized for assay. TBI significantly reduced VEGF, HGF, and IGFBP-6 levels in the eye, while MSC therapy markedly reversed these changes (except VEGF). **(D)** Western blot analysis of IGF-1R, VEGFR2, phosphorylated VEGFR2 (p-VEGFR2), and c-Met in R28 cell lysates. SI reduced receptor expression, while MSC coculture significantly restored it. Densitometric quantification is normalized to β-actin. **(E)** Western blot analysis of downstream signaling proteins in R28 cells. MSC coculture upregulated phosphorylated Akt (p-Akt), Sirtuin 1 (SirT1), and β-catenin in SI-injured cells. Quantified values are normalized to β-actin (p < 0.0001). Abbreviations: HGF, hepatocyte growth factor; IGFBP-4/6, insulin-like growth factor binding protein-4/6; VEGF, vascular endothelial growth factor; IGF-1R, insulin-like growth factor 1 receptor; VEGFR2, vascular endothelial growth factor receptor 2; p-Akt, phosphorylated Akt; SirT1, Sirtuin 1. Data are presented as mean ± SD. *In vitro* experiments (A, D, E) were performed in at least three independent replicates. *In vivo* data (B, C) are from n=8-9 (serum ELISA) or n = 6 (ocular tissues ELISA) animals per group. For original blot images, see the Supplementary Data file.

To assess whether MSC therapy enhances neurotrophic support, we measured the expression of key growth factors, including HGF, IGFBP-4, IGFBP-6, and VEGF. *In vitro*, MSC coculture significantly increased the secretion of these factors in stretch-injured R28 cells compared to SI controls (p < 0.0001 for HGF; p = 0.001 for IGFBP-4; p < 0.0001 for IGFBP-6; p < 0.0001 for VEGF; **Fig. 9A**; original blots shown in **Supplementary Figure S4**). *In vivo*, TBI markedly reduced serum levels of VEGF (p < 0.0001), HGF (p = 0.0072), IGFBP-4 (p = 0.0214), and IGFBP-6 (p = 0.0002), while MSC treatment significantly restored their expression, excepted IGFBP-6 (**Fig. 9B**). Consistent with the serum data, ELISA analysis of ocular tissue lysates revealed that MSC therapy reversed the TBI-induced reduction of HGF and IGFBP-6, but VEGF and IGFBP-4 (**Fig. 9C**). These findings support a role for MSCs in restoring local and systemic neurotrophic signaling following neurotrauma.

We further assessed the expression of downstream signaling molecules in R28, including IGF-1R, VEGFR2, p-VEGFR2, c-Met, p-Akt, Akt, SirT1, and β-catenin, in R28 cells (**Fig. 9D, 9E**). Compared to control cells, stretch injury significantly reduced IGF-1R (p < 0.0001), p-VEGFR2 (p = 0.0458), and β-catenin (p < 0.0001) levels. Notably, MSC coculture significantly restored IGF-1R (p = 0.0358), p-VEGFR2 (p = 0.0476) and β-catenin (p < 0.0001) expression, suggesting that MSCs promote neurotrophic receptor signaling and β-catenin pathway activity. Original immunoblot images for **Fig. 9D-E** are provided in **Supplementary Figures S5 and S6**.

**Figure 10:**
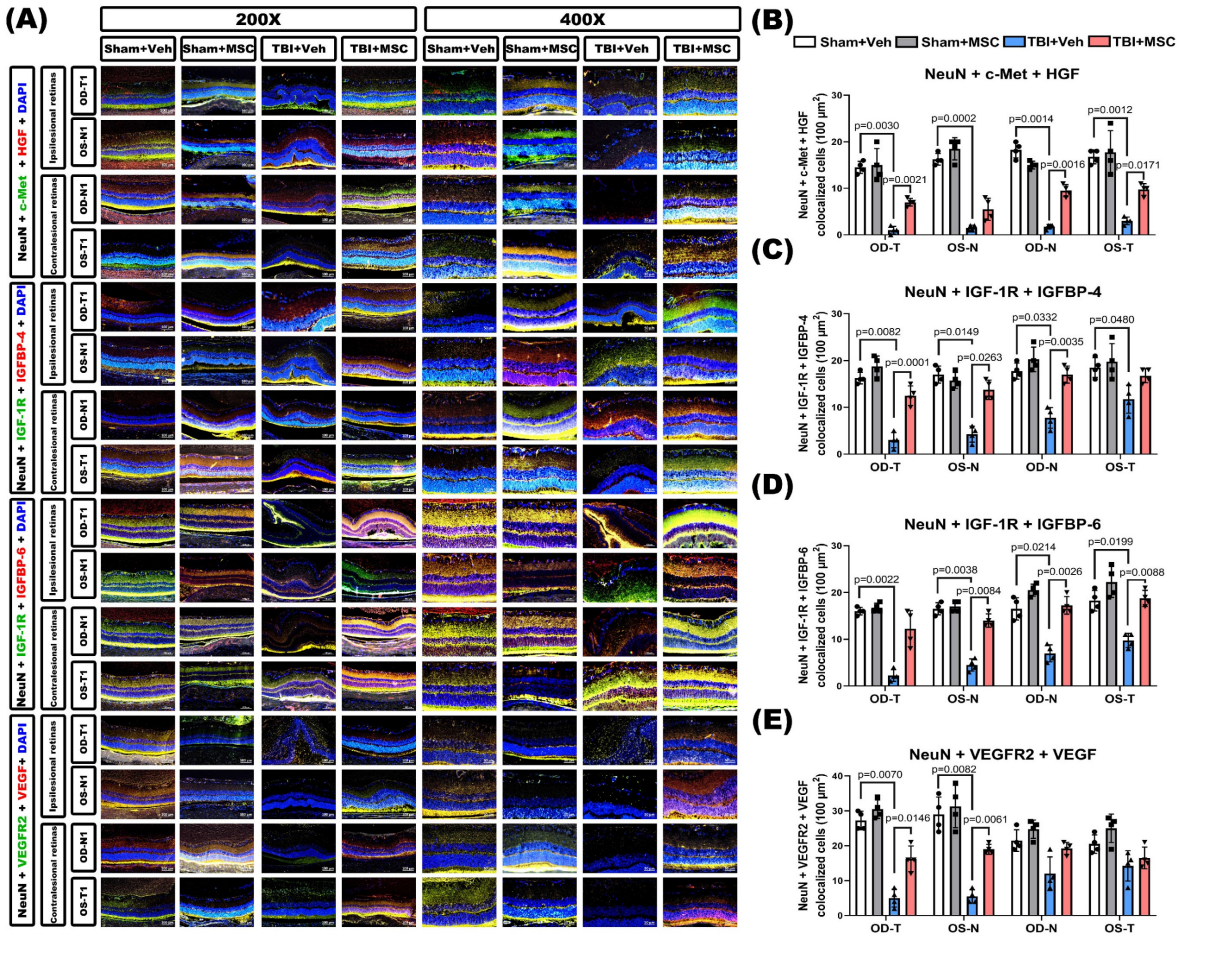
** MSC therapy restores TBI-induced neurotrophic growth factor signaling downregulation in retinal neurons. (A)** Representative immunofluorescence images of retinal sections (400× magnification) from the indicated groups (Sham+Veh, Sham+MSC, TBI+Veh, TBI+MSC). Images show neuronal co-expression of growth factor ligands and receptors. Top panel: Triple staining for NeuN (neuronal marker, gray), c-Met (HGF receptor, green), and HGF (red). Second panel: NeuN, IGF-1R (IGF receptor, green), and IGFBP-4 (red). Third panel: NeuN, IGF-1R (green), and IGFBP-6 (red). Bottom panel: NeuN, VEGFR2 (VEGF receptor, green), and VEGF (red). DAPI (blue) stains nuclei. Retinal regions include ipsilesional OD-T, OS-N and contralesional OD-N, OS-T segments. **(B-E)** Quantification of triple-positive cells (NeuN+ growth factor receptor+ ligand) per retinal section: **(B)** NeuN+c-Met+HGF, **(C)** NeuN+IGF-1R+IGFBP-4, **(D)** NeuN+IGF-1R+IGFBP-6, **(E)** NeuN+VEGFR2+VEGFA. Data were obtained from 6 animals in each group and expressed as mean±SD.

To validate these findings *in vivo*, immunofluorescence staining was performed in retinal sections from TBI rats. TBI markedly downregulated neuronal growth factor signaling, as indicated by reduced colocalization of NeuN with c-Met+HGF, IGF-1R+IGFBP-4, IGF-1R+IGFBP-6, and VEGFR2+VEGF (**Fig. 10**). MSC therapy significantly restored these neuronal signaling interactions, supporting its role in enhancing retinal neurotrophic signaling and neuronal protection following TBI.

## Discussion

TBI is frequently associated with visual disturbances and retinal pathology, both of which can significantly impair cognitive function and reduce quality of life 22. While cortical visual areas are often compromised, increasing evidence indicates that TBI also induces structural and functional changes in the retina, including RGC loss and thinning of the retinal nerve fiber layer (RNFL) 23, 24. These retinal abnormalities may exacerbate visual deficits independently of cortical injury and can persist long after the initial insult. Moreover, retinal changes post-TBI have been shown to mirror early Alzheimer's disease-like pathology, positioning the retina as a sensitive and non-invasive biomarker of central nervous system (CNS) degeneration 25. Given the dual role of the retina as both a contributor to visual processing and a potential indicator of broader neuropathology, addressing post-TBI retinopathy is not only relevant for vision restoration but also for tracking and possibly mitigating progressive neurodegeneration.

In this study, we demonstrated that MSC therapy effectively reversed both neurological and visual dysfunction in a rat model of repeated TBI. Neurological deficits and visual impairments were observed as early as day 7 post-injury and persisted through day 35, underscoring the sustained impact of TBI on central and visual circuits. Intravenous MSC administration significantly improved both sensorimotor and visual performance, preserved retinal structure, and reduced RGC apoptosis. These therapeutic effects were associated with restoring β-catenin expression in retinal neurons, a key molecule involved in synaptic integrity, neuronal survival, and Wnt pathway regulation 26, 27. Taken together with our systemic and ocular growth factor data, these findings support a working model in which repeated TBI disrupts neurotrophic support and β-catenin-dependent survival signalling in both brain and retina, whereas systemically delivered MSCs repair this trophic milieu, stabilize β-catenin, and thereby limit progressive neurodegeneration and functional decline. These results suggest that MSC therapy may represent a viable translational strategy for mitigating vision-related and neurodegenerative consequences of TBI in clinical settings.

To further dissect the mechanisms underlying MSC-mediated retinoprotection, we employed an *in vitro* stretch injury (SI) model using R28 retinal precursor cells. This model recapitulated hallmark features of TBI-induced retinal degeneration, including cell death, mitochondrial dysfunction, oxidative stress, caspase activation, and β-catenin downregulation, closely mirroring the *in vivo* pathology. These parallel responses validate SI as a translationally relevant model 15, 28. Although the SI model does not reproduce the full complexity of secondary injury after TBI, it provides a controlled platform to test whether MSC-derived factors can directly counteract cell-intrinsic stress pathways in retinal neurons that converge on β-catenin loss, mitochondrial damage, and apoptosis, thereby complementing the *in vivo* evidence of systemic trophic restoration. MSC coculture effectively reversed these degenerative changes, supporting the hypothesis that MSCs confer protection via paracrine signaling that rescues injured retinal neurons.

A central finding of this study is the restoration of neurotrophic growth factor signaling in both *in vitro* and *in vivo* models. SI reduced the expression of IGFBP-4, IGFBP-6, and VEGF, as well as their respective receptors, IGF-1R and VEGFR2. These disruptions were similarly observed in the serum and ocular tissues of TBI rats and were reversed by MSC therapy. Immunofluorescence staining further revealed increased colocalization of NeuN-positive neurons with growth factor receptors (c-Met, IGF-1R, and VEGFR2), confirming MSC-mediated reactivation of receptor-ligand neurotrophic signaling pathways. Thus, the convergent *in vivo* and *in vitro* data are consistent with a causal sequence in which MSC-driven restoration of HGF/IGFBP/VEGF signalling in NeuN-positive cells stabilizes β-catenin, alleviates oxidative and autophagic stress, and ultimately reduces RGC death and preserves visual function.

VEGF plays a multifaceted role in retinal health and disease. Beyond its well-known angiogenic effects, VEGF is critical for neuronal survival, synaptic integrity, and Müller glial cell function in the retina 29-31. Studies have shown that VEGF supports photoreceptors and retinal ganglion cells under stress and is essential for maintaining retinal homeostasis 29. However, VEGF is a double-edged sword. While its neuroprotective actions are beneficial, excessive, or unbalanced, VEGF signaling contributes to vascular leakage, edema, and pathological neovascularization, particularly in conditions such as diabetic retinopathy and age-related macular degeneration 32, 33. Therefore, MSC-mediated restoration of VEGF expression, as observed in our study, may enhance its neurotrophic benefits while avoiding the harmful angiogenic consequences, possibly through co-modulation with β-catenin and IGF signaling. This highlights the importance of maintaining a tightly regulated VEGF signaling environment for therapeutic success in TBI-induced retinal degeneration.

IGF signaling also plays a fundamental role in retinal repair. Activation of IGF-1R supports neuronal survival and regeneration through PI3K/Akt and MAPK pathways 34, 35. IGFBPs, particularly IGFBP-4 and IGFBP-6, fine-tune this signaling by modulating IGF bioavailability, enhancing its stability, and facilitating localized delivery 35. In our study, MSCs restored the downregulated expression of IGFBPs and IGF-1R in both SI-injured R28 cells and TBI retinas, suggesting coordinated reactivation of this neurotrophic axis.

The HGF/c-Met pathway further supports retinal repair. HGF has been shown to exert anti-apoptotic and anti-inflammatory effects in retinal neurons and glia 36, 37. Our results demonstrated that MSCs restored HGF levels in both serum and ocular tissue, alongside increased c-Met expression in NeuN-positive neurons. This aligns with previous studies showing that MSC-secreted HGF plays a critical role in CNS and retinal repair 38, 39. Together, these growth factor pathways form a coordinated neuroprotective network promoted by MSCs.

Beyond trophic signaling, MSCs also modulated critical cell death pathways. SI activated autophagy (increased LAMP2, LC3B-II) and apoptosis (elevated caspase-3 and -9), consistent with findings in models of retinal ischemia and glaucoma 40. MSCs reversed these changes, restored mitochondrial membrane potential, and reduced intracellular ROS levels. This suggests that MSCs alleviate oxidative stress, recalibrate autophagic flux, and inhibit caspase-driven cell death.

At the molecular level, β-catenin signaling emerged as a key integrator of these protective mechanisms. Its stabilization through GSK-3β inhibition promotes neuronal survival, while caspase-3-mediated degradation impairs repair 41. MSC therapy restored β-catenin expression while reducing caspase-3 activation, suggesting that MSCs promote β-catenin-dependent survival pathways. Within this framework, β-catenin functions not merely as a marker of neuronal viability but as a downstream integrator of MSC-induced trophic signalling from VEGF, HGF, and IGF pathways, linking paracrine factor restoration to reduced apoptosis and improved retinal structure. Importantly, VEGFR and IGF-1R signaling are known to cross-talk with β-catenin 42, indicating a converging axis of MSC-mediated neuroprotection.

Although pharmacological activation of individual pathways such as IGF-1R 34, 35 or VEGFR 43-46 may replicate certain components of MSC-mediated neuroprotection, MSCs likely offer broader and more integrated therapeutic benefits through their multifactorial mechanisms of action. Beyond growth factor secretion, MSCs modulate immune responses, attenuate inflammation, regulate autophagy, and release extracellular vesicles enriched with microRNAs and bioactive proteins that influence neuronal and glial cell function 47, 48. These coordinated effects facilitate a more comprehensive neuroprotective and neuroregenerative environment, which is challenging to replicate using single-agent pharmacotherapy. Future studies should explore whether targeted pharmacological stimulation of IGF-1R, VEGFR2, or β-catenin pathways alone or in combination can approximate the efficacy of MSC therapy in TBI-induced retinal degeneration. Comparative studies of MSCs versus pharmacological mimetics could help define the most clinically viable and biologically grounded strategies for neurorepair.

### Limitations and future directions

Several limitations must be noted. First, we did not include flash visual evoked potentials (FVEPs), which are critical for assessing functional visual pathway integrity; incorporating FVEPs in future studies will help correlate histological and behavioral outcomes with electrophysiological recovery. Second, although we assessed neurological improvement using the mNSS, which encompasses movement, reflex, sensation, and balance, we did not directly evaluate cognitive function in this study; nonetheless, our previous work has demonstrated that MSC therapy significantly attenuates cognitive deficits and white matter disintegration in a repeated TBI model over a prolonged period (from 1 month to 9 months post-injury) 19, so future studies should combine cognitive assessments with retinal and visual evaluations to better understand how MSC therapy affects both visual and cognitive recovery. Third, we did not perform* in vivo* tracking of intravenously administered MSCs and therefore cannot determine their homing or long-term engraftment in the retina or brain; our mechanistic interpretation is based on paracrine restoration of neurotrophic signalling rather than direct cell replacement. Moreover, we did not carry out loss-of-function experiments (for example, pharmacological blockade of c-Met, IGF-1R, VEGFR2, or β-catenin inhibition) to definitively prove causality within the proposed pathway, so the mechanistic model presented here should be regarded as hypothesis-generating and will require future validation. Fourth, we did not directly compare whole-cell MSC therapy with MSC-derived secretome or extracellular vesicle (EV)-based approaches, which may offer distinct safety and standardization advantages in long-term TBI models. Fifth, although we identified restoration of β-catenin, VEGF, and IGF-related signalling after MSC treatment, the specific molecular mediators responsible for these effects (for example, defined MSC-derived exosomes, microRNAs, or protein cargo) remain to be clarified; future work should dissect which secretome components are necessary and sufficient to reproduce the retinal protection observed here and whether pharmacological or cell-free strategies can approximate the efficacy of MSC therapy. Finally, although our study focused on retinal structural and molecular changes, we did not directly examine immune cell or inflammatory responses within the retina after TBI. Our previous work 19 demonstrated that MSC therapy reduces the accumulation of TNF-α-positive microglia and attenuates gray matter damage post-TBI, highlighting the importance of microglial modulation in MSC-mediated neuroprotection; given the established role of microglia in retinal neuroinflammation and degeneration 49 and prior evidence that MSC-derived secretome and extracellular vesicles can modulate microglial activation in CNS and retinal injury models 50-52, future studies of TBI-induced retinal injury should build on this work to determine whether similar MSC-microglia interactions and inflammatory pathways are engaged along the visual pathway after neurotrauma.

## Conclusion

Our findings demonstrate that MSC therapy ameliorates TBI-induced retinal and neurological deficits by restoring neurotrophic signaling pathways, reducing oxidative and apoptotic stress, and preserving neuronal integrity. The coordinated activation of β-catenin, VEGF, IGFBP, and HGF signaling cascades appears central to these effects. These results highlight the potential of MSCs as a multifactorial therapeutic platform for treating TBI-associated retinal degeneration and warrant further exploration in translational and clinical contexts.

## Supplementary Material

Supplementary figures.

## Figures and Tables

**Table 1:** Antibodies and commercial kits used in the biochemical assay

Antibody/kit	Titre	Company	Catalog #	Purpose
Caspase 3	1:2000	Cell Signaling Technology	9662	WB
Caspase 9	1:2000	Cell Signaling Technology	9508	WB
LC3B-I/II	1:2000	Cell Signaling Technology	2775	WB
pc-Met	1:2000	Cell Signaling Technology	3126	WB
C-Met 1454Y	1:2000	Abcam	Ab51067	WB
IGF-1R	1:2000	Cell Signaling Technology	3027	WB
VEGFR2	1:1000	Cell Signaling Technology	9698	WB
p-VEGFR2	1:1000	Abcam	Ab5473	WB
Akt	1:2000	Cell Signaling Technology	9272	WB
p-Akt (Ser473)	1:2000	Abcam	Ab81283	WB
β-catenin	1:2000	Cell Signaling Technology	9562	WB
LAMP2	1:5000	Abcam	ab125068	WB
β-actin	1:15000	Santa Cruz	SC-47778	WB
Anti-rabbit IgG, HRP-linked Antibody	1:2000	Cell Signaling Technology	7074	WB
Anti-mouse IgG, HRP-linked Antibody	1:2000	Cell Signaling Technology	7076	WB
DAPI	1:50000	Invitrogen	D1306	IF
β3-tubulin	1:200	Abcam	ab18207	IF
MAP-2	1:200	Santa Cruz	SC-74421	IF
β-catenin	1:200	Cell Signaling Technology	8480	IF
NeuN	1:200	Merck	MAB377	IF
C-Met	1:200	Abcam	ab51067	IF
HGF	1:200	Abcam	ab216623	IF
IGF-1R	1:200	Elabscience	E-AB-60038	IF
IGFBP-4	1:200	Abcam	ab239512	IF
IGFBP-6	1:200	Bioss	BS-4064R	IF
VEGFR2	1:200	Abcam	ab39638	IF
VEGF	1:200	Abcam	ab1316	IF
Goat anti-mouse IgG (Alexa Fluor 488)	1:400	Invitrogen	A11029	IF
Goat anti-rabbit IgG (Alexa Fluor 488)	1:400	Invitrogen	A11034	IF
Goat anti-mouse IgG (Alexa Fluor 568)	1:400	Invitrogen	A11004	IF
Goat anti-rabbit IgG (Alexa Fluor 568)	1:400	Invitrogen	A11011	IF
MitoScreen (JC-1) kit		BD Biosciences	551302	Flow cytometry
ROS-ID^®^ Total ROS/Superoxide detection kit		ENZO Life Sciences	ENZ-51010	Flow cytometry
TUNEL Apoptosis Assay Kit		BioTnA Biotech	TAAP01F-100	IF
Luxol Fast Blue Stain Kit (Myelin Stain)		Abcam	ab150675	IHC
Rat VEGF-A Kit		Aviva Systems Biology	OKRC01482	ELISA
Rat HGF Kit		Aviva Systems Biology	OKEH00072	ELISA
Rat IGFBP-4 Kit		Aviva Systems Biology	OKEH00087	ELISA
Rat IGFBP-6 Kit		Aviva Systems Biology	OKEH00355	ELISA

**Abbreviations**: WB, Western blot; IF, Immunofluorescence; IHC, immunohistochemistry; ELISA, Enzyme-linked Immunosorbent Assay.

## Data Availability

The authors confirm that the data supporting the findings of this study are all available within the article.

## Abbreviations

BDNF: brain-derived neurotrophic factor
DCFH-DA: dichlorodihydrofluorescein diacetate
FVEPs: flash visual evoked potentials
GCC: ganglion cell complex
GDNF: glial cell line-derived neurotrophic factor
HGF: hepatocyte growth factor
IGF1R: insulin-like growth factor 1 receptor
IGFBP-4: insulin-like growth factor binding protein-4
IGFBP-6: insulin-like growth factor binding protein-6
INL: inner nuclear layer
IPL: inner plexiform layer
IS/OS: photoreceptor inner segment/outer segment
LAMP2: lysosome-associated membrane protein 2
LC3B-II: microtubule-associated protein 1 light chain 3β-II
LFP: lateral fluid percussion
LGN: lateral geniculate nucleus
MAP2: microtubule-associated protein 2
mNSS: modified neurological severity score
MSC: mesenchymal stem cell
NeuN: neuronal nuclear protein
NFL/GCL: nerve fiber layer and ganglion cell layer
ONL: outer nuclear layer
OPL: outer plexiform layer
RGC: retinal ganglion cell
RND: retinal neurodegenerative disease
ROS: reactive oxygen species
RPE: retinal pigment epithelium
RTD: retrograde transsynaptic degeneration
SC: superior colliculus
SI: stretch injury
TBI: traumatic brain injury
TUNEL: terminal deoxynucleotidyl transferase (TdT) dUTP nick end labeling
VEGF: vascular endothelial growth factor
VEGFR2: vascular endothelial growth factor receptor 2
Veh: vehicle
