# Supplementary material for: Stem cells ameliorate neurotrauma-induced visual disturbances and retinal degeneration via broad normalization of β-catenin-related signaling
Source: Int J Med Sci. 2026 Jan 23;23(3):758–79. doi: 10.7150/ijms.123975 (PMC12964567; doi:10.7150/ijms.123975)
Supplement: Supplementary file 1 — Supplementary figures. [file ijmsv23p0758s1.pdf]

Supplementary file

Stem cells ameliorate neurotrauma-induced visual disturbances and retinopathy via broad normalization of the β-catenin-related signaling pathway

Shu-Chun Kuo[1,2,*], Chung-Hsin Tseng[3,4], Suan Hwang[1], Chia-Yi Lee[1], Ting-Feng Wu[4], Pi-Yu Chao[3], Anthony Lu[3], Ching-Ping Chang[3,*], Chung-Ching Chio[5,*]

[1]Department of Ophthalmology, Chi Mei Medical Center, Tainan, Taiwan

[2]Department of Optometry, Chung Hwa University of Medical Technology, Tainan, Taiwan

[3]Department of Medical Research, Chi Mei Medical Center, Tainan 710, Taiwan

[4]Department of Biotechnology and Food Technology, Southern Taiwan University of Science and Technology, Tainan 710, Taiwan

[5]Division of Neurosurgery, Department of Surgery, Chi Mei Medical Center, Tainan 717, Taiwan

***Correspondence to:**

Shu-Chun Kuo

Department of Ophthalmology, Chi Mei Medical Center.

Department of Optometry, Chung Hwa University of Medical Technology. Address: No. 901, Zhonghua Rd, Yongkang District, Tainan City 710, Taiwan. E-mail: ophkuo@gmail.com

Ching-Ping Chang, Ph.D., Department of Medical Research, Chi Mei Medical Center. Address: No. 901, Zhonghua Rd, Yongkang District, Tainan City 710, Taiwan. E-mail: jessica.cpchang@gmail.com or a50831@mail.chimei.org.tw

Chung-Ching Chio, M.D., Division of Neurosurgery, Department of Surgery, Chi Mei Medical Center. Address: No. 901, Zhonghua Rd, Yongkang District, Tainan City 710, Taiwan. E-mail: chiocc@ms28.hinet.net

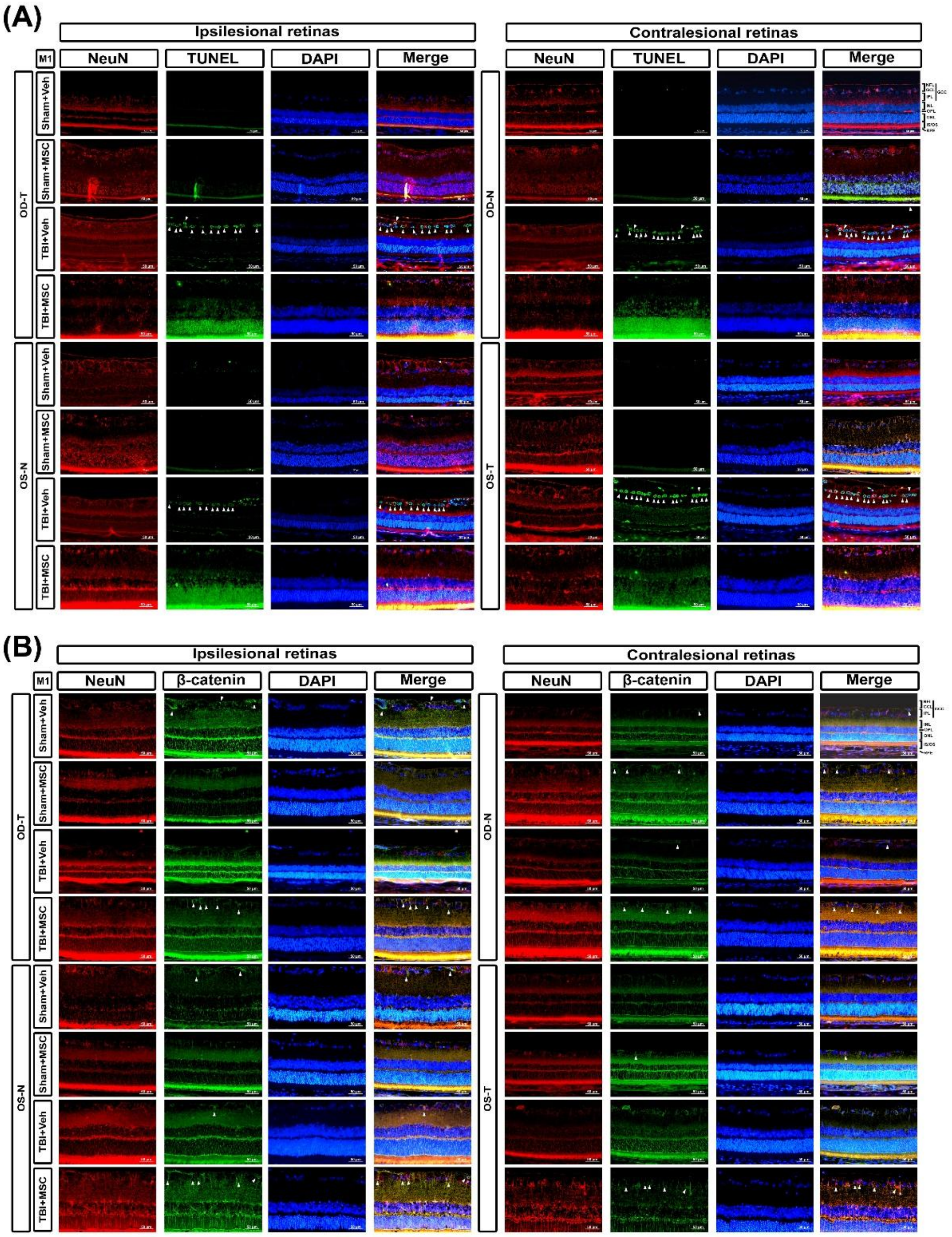

Supplementary Figure S1: Single-channel fluorescence images corresponding to Figure 4. Representative single-channel immunofluorescence images of NeuN (neuronal marker, red), TUNEL (apoptosis marker, green), β-catenin (green), and DAPI (nuclear marker, blue) from the retina of Sham+Veh, Sham+MSC, TBI+Veh, and TBI+MSC rats. These images provide a clearer visualization of individual fluorescence signals before merging in Figure 4A and B, ensuring accurate interpretation of neuronal apoptosis in the ganglion cell complex (GCC). Scale bar = 50 µm.

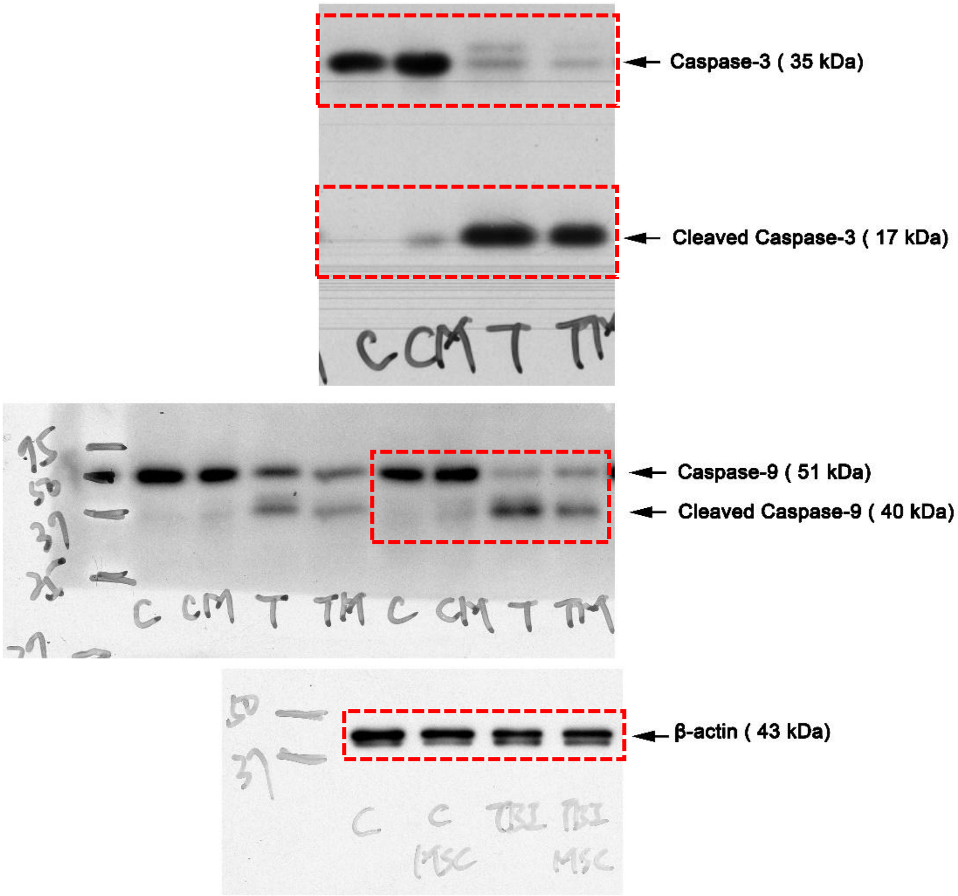

Supplementary Figure S2: Full-length Western blots corresponding to Figure 7C. Each lane is labeled by handwritten abbreviations on the original films: C = control (R28 without stretch injury), CM = C+MSC (control plus MSC coculture), T = SI (stretch-injured R28 cells), and TM = SI+MSC (stretch-injured R28 cells cocultured with MSCs). Red dashed boxes indicate the regions that were cropped and shown in the main figure; no non-adjacent lanes were spliced or rearranged, and any brightness/contrast adjustments were applied uniformly across the entire blot.

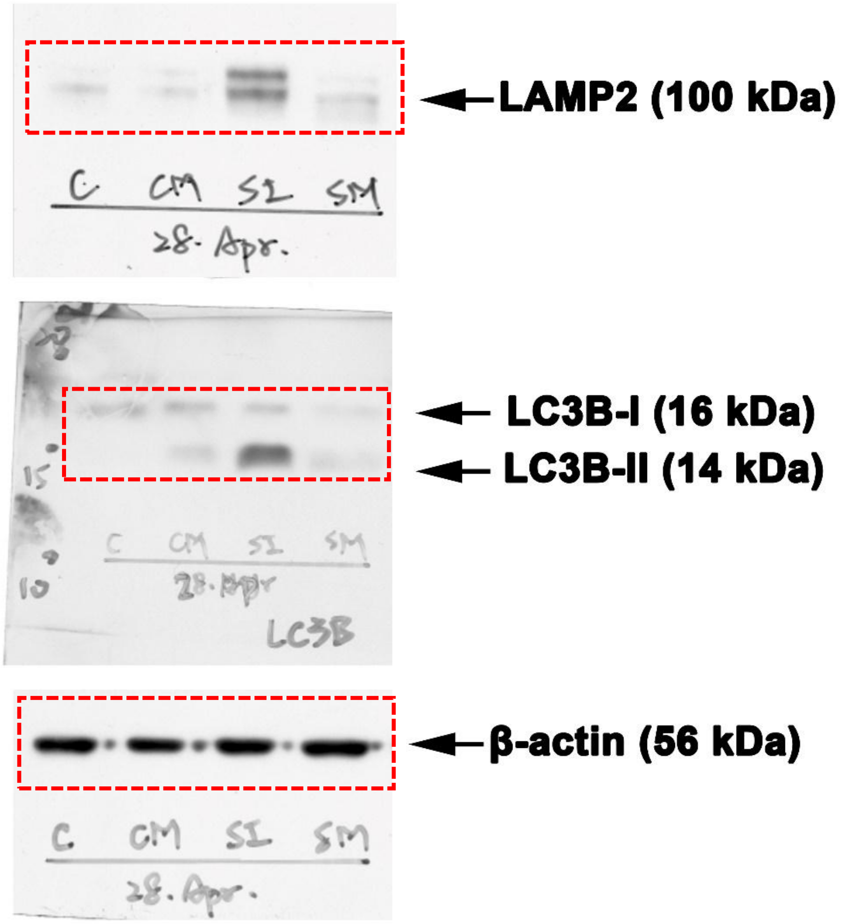

Supplementary Figure S3: Full-length Western blots corresponding to Figure 8E. Each lane is labeled by handwritten abbreviations on the original films: C = control (R28 without stretch injury), CM = C+MSC (control plus MSC coculture), T = SI (stretch-injured R28 cells), and TM = SI+MSC (stretch-injured R28 cells cocultured with MSCs). Red dashed boxes indicate the regions that were cropped and shown in the main figure; no non-adjacent lanes were spliced or rearranged, and any brightness/contrast adjustments were applied uniformly across the entire blot.

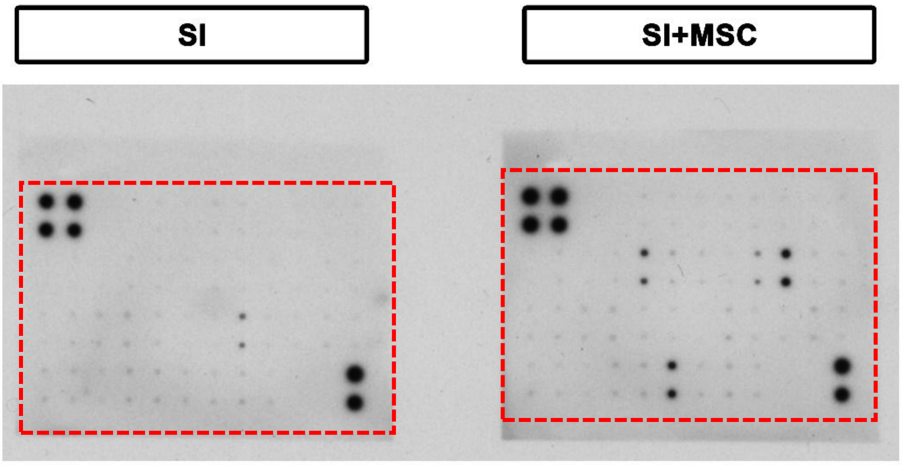

Supplementary Figure S4: Full-length membranes for the growth factor antibody array shown in Figure 9A. Each membrane corresponds to conditioned medium collected from stretch-injured R28 cells without MSC coculture (SI) or with MSC coculture (SI+MSC), as indicated by the handwritten labels on the original films. Labels printed on the right of each X-ray film indicate the capture antibodies spotted on the membrane. Red dashed boxes outline the regions that were cropped and presented in the main figure; no non-adjacent areas were spliced or rearranged, and any brightness/contrast adjustments were applied uniformly to the entire membrane.

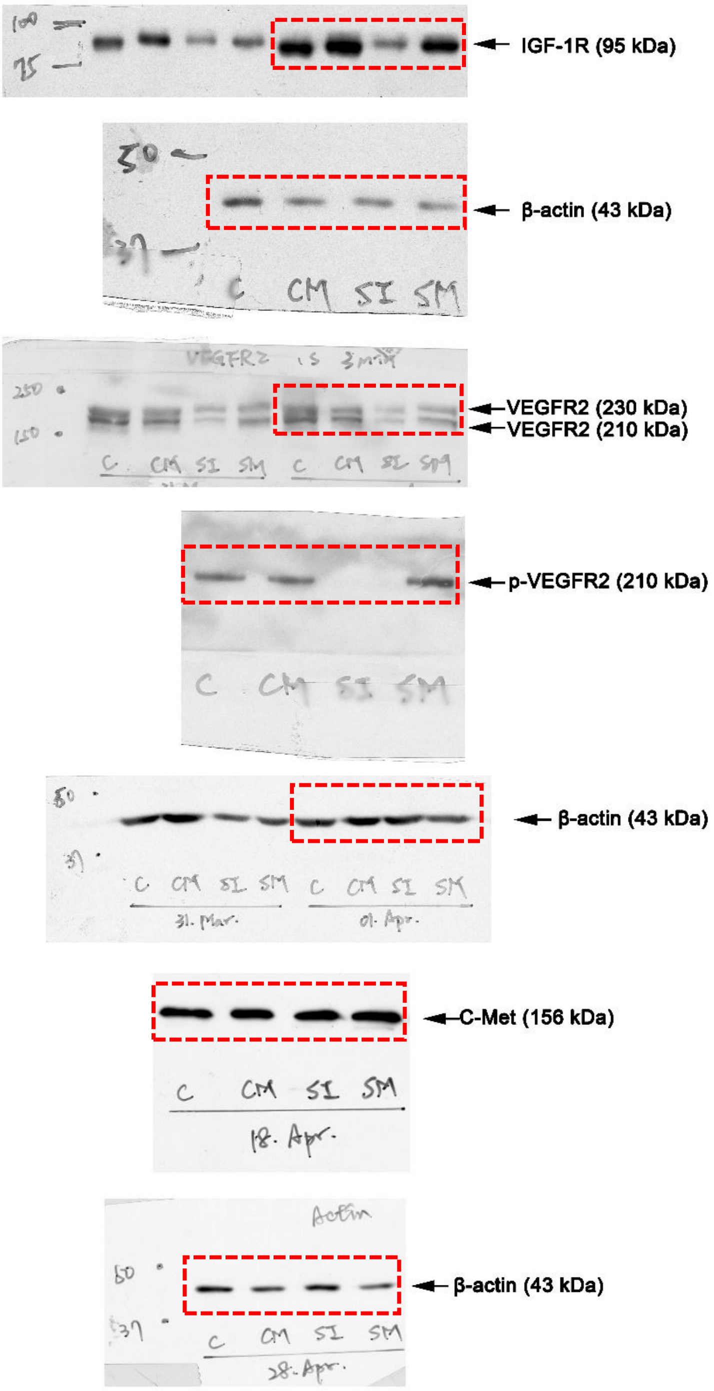

Supplementary Figure S5: Full-length Western blots corresponding to Figure 9D. Each lane is labeled by handwritten abbreviations on the original films: C = control (R28 without stretch injury), CM = C+MSC (control plus MSC coculture), T = SI (stretch-injured R28 cells), and TM = SI+MSC (stretch-injured R28 cells cocultured with MSCs). Red dashed boxes indicate the regions that were cropped and shown in the main figure; no non-adjacent lanes were

spliced or rearranged, and any brightness/contrast adjustments were applied uniformly across the entire blot.

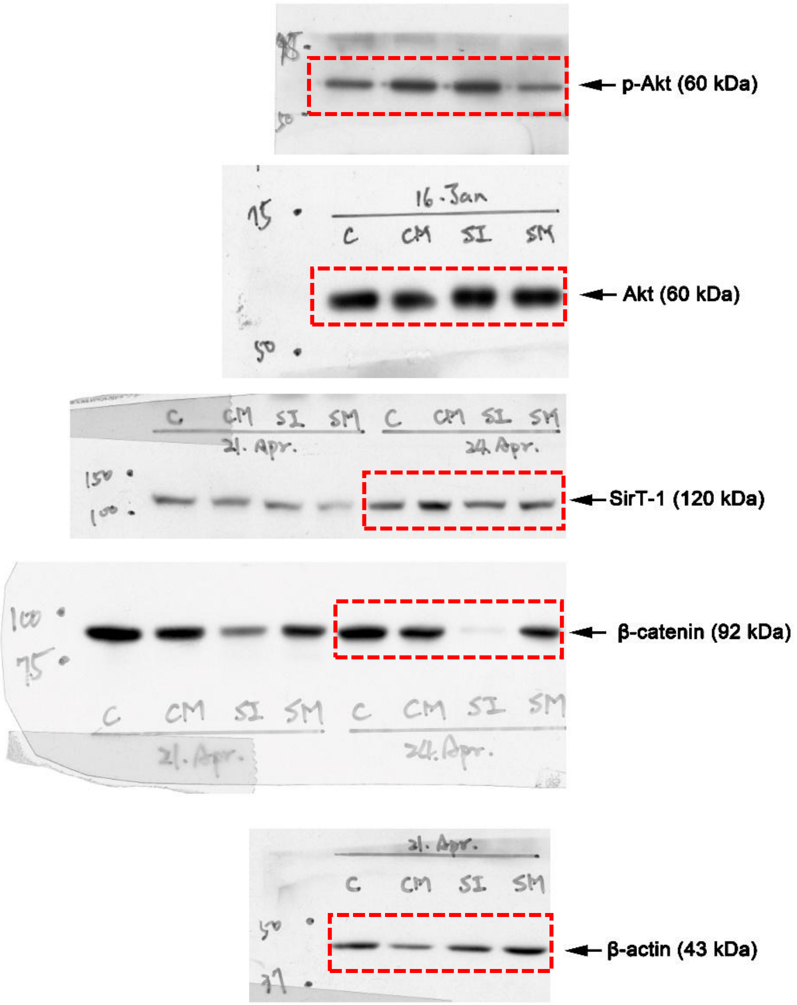

Supplementary Figure S6: Full-length Western blots corresponding to Figure 9E. Each lane is labeled by handwritten abbreviations on the original films: C = control (R28 without stretch injury), CM = C+MSC (control plus MSC coculture), T = SI (stretch-injured R28 cells), and TM = SI+MSC (stretch-injured R28 cells cocultured with MSCs). Red dashed boxes indicate the regions that were cropped and shown in the main figure; no non-adjacent lanes were spliced or rearranged, and any brightness/contrast adjustments were applied uniformly across the entire blot.
